# Reactive Sulfur Compounds in the Fight against COVID-19

**DOI:** 10.3390/antiox11061053

**Published:** 2022-05-26

**Authors:** Małgorzata Iciek, Anna Bilska-Wilkosz, Michał Kozdrowicki, Magdalena Górny

**Affiliations:** Chair of Medical Biochemistry, Medical College, Jagiellonian University, 31-034 Kraków, Poland; mbbilska@cyf-kr.edu.pl (A.B.-W.); michal.kozdrowicki@student.uj.edu.pl (M.K.); magdalena.gorny@uj.edu.pl (M.G.)

**Keywords:** SARS-CoV-2, spike protein, hydrogen sulfide, glutathione, N-acetylcysteine, lipoic acid, erdosteine, ergothioneine

## Abstract

The SARS-CoV-2 coronavirus pandemic outbreak in 2019 resulted in the need to search for an effective and safe strategy for treating infected patients, relieving symptoms, and preventing severe disease. SARS-CoV-2 is an RNA virus that can cause acute respiratory failure and thrombosis, as well as impair circulatory system function. Permanent damage to the heart muscle or other cardiovascular disorders may occur during or after the infection. The severe course of the disease is associated with the release of large amounts of pro-inflammatory cytokines. Due to their documented anti-inflammatory, antioxidant, and antiviral effects, reactive sulfur compounds, including hydrogen sulfide (H_2_S), lipoic acid (LA), N-acetylcysteine (NAC), glutathione (GSH), and some other lesser-known sulfur compounds, have attracted the interest of scientists for the treatment and prevention of the adverse effects of diseases caused by SARS-CoV-2. This article reviews current knowledge about various endogenous or exogenous reactive sulfur compounds and discusses the possibility, or in some cases the results, of their use in the treatment or prophylaxis of COVID-19.

## 1. SARS-CoV-2

Coronavirus disease 2019 (COVID-19) induced by severe acute respiratory syndrome coronavirus 2 (SARS-CoV-2), was first discovered in an outbreak of respiratory disease cases in Wuhan City, China, in 2019 and quickly spread across the world causing a global pandemic. The clinical symptoms of COVID-19 vary from mild respiratory complaints to severe disease with pneumonia and inflammatory disease, acute respiratory failure, multiorgan failure, or death [1]. It has been documented that some people, including men, older patients, and patients with comorbidities (e.g., cardiovascular disease, diabetes mellitus and obesity, chronic respiratory disease, and immune deficiency), are more prone to developing a severe course of the disease requiring hospitalization [2]. Current strategies to treat COVID-19 disease include antiviral drugs (e.g., hydroxychloroquine and remdesivir), antipyretics, non-steroidal anti-inflammatory drugs, mechanical ventilation, and oxygen supplementation when needed [3,4]. Along with many efforts to develop vaccines, there is also a need to identify new drugs that could relieve the symptoms of COVID-19, attenuate its side effects, and which also have the potential to reduce post-COVID-19 complications. Moreover, it would be very useful to find a prognostic biomarker that can predict the disease severity in a course of COVID-19. Some sulfur compounds presented in this review are of interest for both these applications. To discuss the therapeutic and preventive strategies and to identify the patients at higher risk of a serious or fatal disease, a better understanding of the pathogenesis of the disease would be helpful.

**Figure 1:**
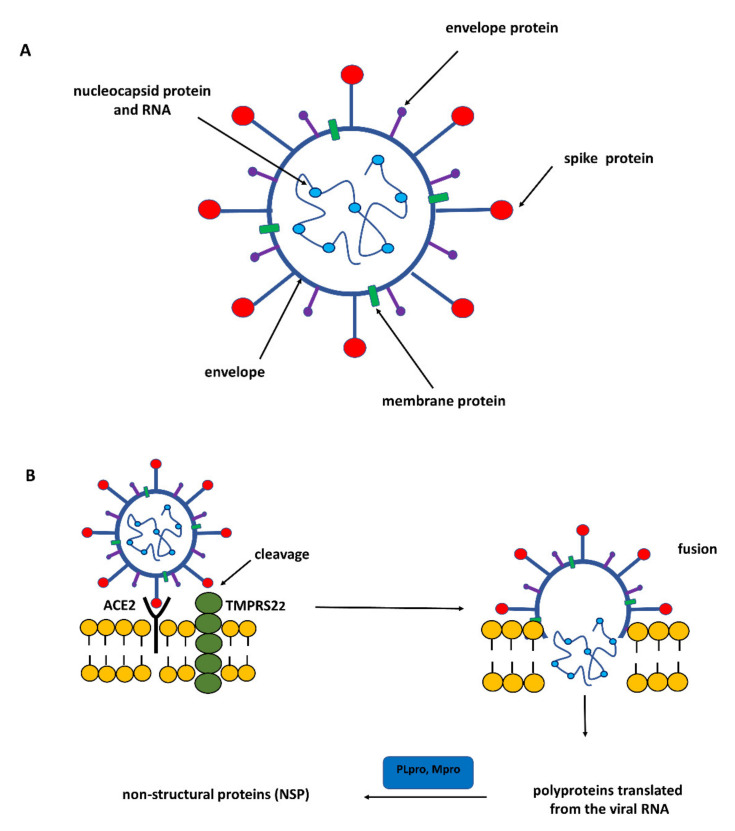
(**A**) Schematic representation of the structure of SARS-CoV-2. (**B**) The infection of the host cell by SARS-CoV-2. TMPRSS2—a transmembrane serine protease 2 that cleaves a dimer of subunits S1/S2 of spike protein making it possible to recognize the angiotensin-converting enzyme 2 (ACE2) receptor on host cells. After fusion of the virus with the host cell, 3-chymotrypsin-like protease called main protease (Mpro) and papain-like protease (PLpro) play a pivotal role in mediating the virus’ replication and transcription by cleaving polyproteins translated from the viral RNA to non-structural proteins (NSP) that are crucial for genome replication and coronavirus virion production.

**Figure 2:**
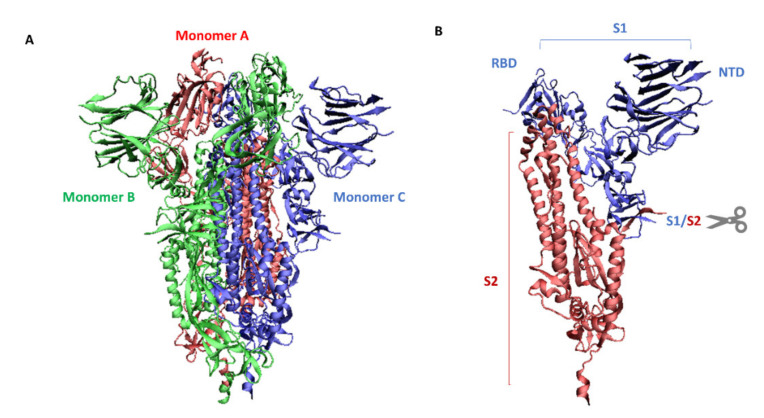
Structure of the SARS-CoV-2 spike protein (PDB: 6VXX). (**A**) The S protein has a trimeric structure and its three monomers are colored in red, green, and blue. (**B**) Each monomer of spike protein consists of two subunits, S1 and S2. S1 subunit has two domains: RBD—receptor-binding domain and NTD—N-terminal domain. RBD domain is responsible for recognizing and binding to the angiotensin-converting enzyme 2 (ACE2) receptor on the host cell surface; NTD domain is involved in the initial binding of the virus to cells. Cleavage of S1/S2 dimer by TMPRSS2 (transmembrane serine protease 2) activates spike protein leading to the host cell infection.

The genome of SARS-CoV-2 belonging to the coronavirus family is a single-stranded positive-sense RNA (+ssRNA) that is larger than other RNA viruses. Structural proteins associated with the genome envelope include a glycoprotein called the S protein (spike protein, S), a membrane protein (M), an envelope protein (E), and a nucleocapsid protein with RNA (Figure 1) [5]. A key, critical role in the process of the infection of host cells by SARS-CoV-2 is played by the large spike protein because it allows the virus to enter the cell and infect it [6,7]. From this point of view, the spike protein is an attractive target for antiviral intervention. The S protein is a trimeric protein and each protomer is made up of two subunits, S1 and S2. The S1 subunit consists of two domains, one of which is the receptor-binding domain (RBD) and is responsible for recognizing and binding to the angiotensin-converting enzyme 2 (ACE2) receptor on the host cell’s surface; the other is the N-terminal domain (NTD) and is involved in the initial binding of the virus to the cells [8]. The S2 subunit, in turn, plays an important role in viral and host cell fusion and the internalization by endocytosis in respiratory epithelial cells. In its native form (as a dimer of two subunits S1 and S2), the S protein is inactive, whereas during infection, it is cleaved into the S1 and S2 subunits by host proteases, and this process is a fundamental step in the fusion process [9] (Figure 2).

**Figure 3:**
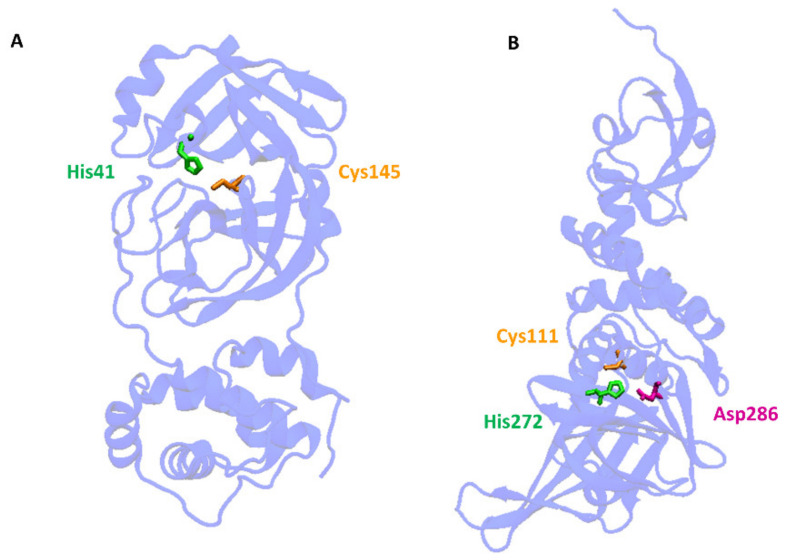
Structures of two pivotal SARS-CoV-2 proteases: (**A**) main protease (Mpro) called also 3-chymotrypsin-like protease (3CLpro) (PDB: 6LU7) and (**B**) papain-like protease (PLpro) (PDB: 6WUU). Catalytic center is formed by Cys145 and His41 in Mpro and by Cys111, His272, and Asp286 in PLpro.

Cleavage of the S protein occurs mainly under the influence of a specific transmembrane serine protease 2 (TMPRSS2) found in the cell membranes in various tissues including the human respiratory tract [10,11,12]. In the case of SARS-CoV-2, to enter host cells the virus recognizes the angiotensin-converting enzyme 2 (ACE2) receptor on the host cells via the receptor-binding domain (RBD). Taking into account the role of ACE2 and TMPRSS2 in the development of infection, it can be concluded that by influencing the activity of these proteins it is possible to limit the risk of the virus entering the host cells and spreading the infection. ACE2 is a multi-tissue transmembrane protein that catalyzes the conversion of proatherosclerotic angiotensin II to angiotensin 1–7 with anti-inflammatory and vasodilating properties [13]. The internalization of ACE2 by SARS-CoV-2 can lead to an increase in angiotensin II concentration, which aggravates the lung damage initiated by the virus [14] (Figure 1). In turn, two crucial cysteine proteases play a pivotal role in mediating the replication and transcription of SARS-CoV-2, namely the main protease (Mpro) called also 3-chymotrypsin-like protease (3CLpro), and the papain-like protease (PLpro) [15]. Both proteases attract a great deal of attention because their activity is critical for the virus’ lifecycle. They cleave polyproteins translated from the viral RNA yielding non-structural proteins that are crucial for genome replication and coronavirus virion production (Figure 1). Mpro is an ideal target for antiviral drug design due to its highly conserved structure in different coronavirus strains and the absence of its functional analogs in the human proteome. Mpro hydrolyzes the viral polyprotein at more than 11 conserved sites. It is a homodimer with a highly conserved active site divided into four (sub)sites (S1–S4). The Cys145 and His41 residues in the S1 (sub)site form the catalytic dyad and play a key role in the activity of Mpro [16]. The formation of a covalent linkage of potential inhibitors with the Cys145 residue creates an opportunity for antiviral activity [17]. On the other hand, PLpro, the second important SARS-CoV-2 protease, has a catalytic triad consisting of Cys112, His273, and Asp287 in its active site (Figure 3). PLpro cleavages the viral polypeptide into functional non-structural proteins and takes up ubiquitin (Ub), thereby suppressing the host’s anti-viral reactions [15]. Therefore, both cysteine proteases, Mpro and PLpro, are equally important for the viral lifecycle and have been intensively studied for their use as crucial targets for drug development.

Another protein gasdermin D (GSDMD) is also the focus of scientists’ attention in the context of COVID-19. It belongs to the gasdermin family and serves as a specific substrate for inflammatory caspase-1. Cleavage of GSDMD by caspase-1 leads to cell membrane permeability, leakage of the cell contents, and finally to programmed cell death known as pyroptosis [18]. It has been documented that cleavage of GSDMD and pyroptosis were inhibited in human monocytes of patients with SARS-CoV-2 [19].

The inflammasomes are critical components of the immune system that regulate the activation of caspase-1 and induce inflammation in response to infectious factors. Among other inflammasomes, NLRP3 plays an important role in mediating caspase-1 activation and the secretion of proinflammatory cytokines in response to microbial pathogens. It has been reported that SARS-CoV-2 involves the inflammasomes in human monocytes, both in patients infected experimentally as well as those with severe COVID-19 [20].

All these aspects combined with the entry of SARS-CoV-2 into the host cells (spike protein, ACE2), the viral replication and transcription (cysteine proteases), and the cellular immune response (inflammasome and GSDMD) are explored in the context of preventing the spread of the virus and finding effective drugs that can relieve the symptoms of the disease and will protect patients from severe illness. In this aspect, some sulfur compounds including hydrogen sulfide (H_2_S), lipoic acid (LA), N-acetylcysteine (NAC), glutathione (GSH), disulfiram (DSF), erdosteine, and ergothioneine (ET) have aroused the interest of scientists due to their well-documented anti-inflammatory, antioxidant, and antiviral effects. H_2_S has been recognized as the third gasotransmitter that is protective against oxidative stress and has a modulatory role. NAC is regarded mainly as a precursor of cysteine and glutathione and in this way, it exerts antioxidant effects. Moreover, the beneficial action of NAC has been documented in a wide range of human diseases. GSH is the main endogenous low-molecular-weight thiol, which has antioxidant properties. A decrease in the level of GSH was observed in many pathologies including COVID-19. DSF is a well-known inhibitor of aldehyde dehydrogenase that can also inhibit other enzymes with cysteine residues, which is responsible for a broad spectrum of its pharmacological activity. LA is an endogenous sulfur compound and strong antioxidant used clinically in some pathologies including diabetes, cardiovascular disorders, and inflammation. Erdosteine is regarded mainly as a mucolytic agent, however, it also possesses many other properties including anti-inflammatory action. On the other hand, naturally occurring ET, besides its antioxidant properties, also has anti-inflammatory potential and its decreased levels have been observed in some diseases. Taking into account the antioxidant, anti-inflammatory, and antiviral effects of the mentioned sulfur compounds, it is not surprising that they can be helpful in relieving the severe symptoms of COVID-19. The mechanisms of their actions are mainly due to the possibility of the modification of cysteine residues in the target proteins playing a key role in viral infection, replication, and transcription. Moreover, they affect cellular immune responses through a reduction of proinflammatory and an elevation of anti-inflammatory cytokines.

## 2. H_2_S and SARS-CoV-2

**Figure 4:**
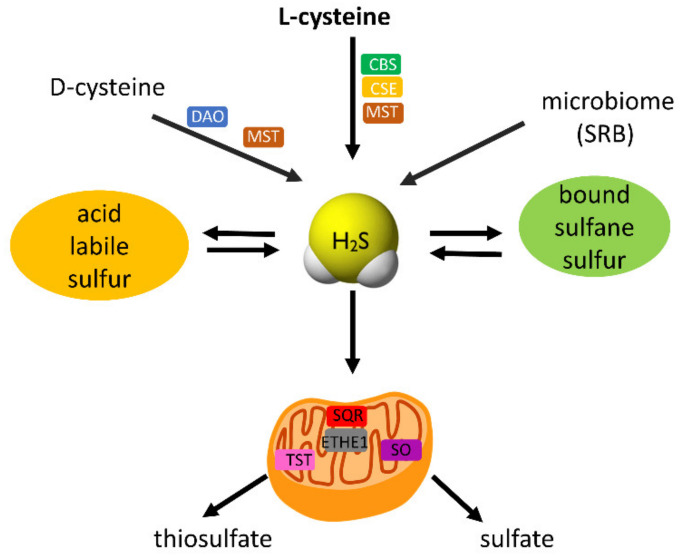
Synthesis and mitochondrial catabolism of H_2_S. H_2_S is synthesized in most tissues from L-cysteine in reactions catalyzed by cystathionine β-synthase (CBS), cystathionine γ-lyase (CSE), and 3-mercaptopyruvate sulfurtransferase (MST). In peroxisomes, H_2_S can be formed from D-cysteine with participation of D-amino acid oxidase (DAO) and then with MST. Another source of H2S in humans is derived from microbiome-containing sulfate-reducing bacteria (SRB). H_2_S coexists in biological conditions in equilibrium with a pool of reactive sulfur, namely bound sulfane sulfur (mainly persulfides and polysulfides) and acid-labile sulfur (mainly iron–sulfur clusters). Compounds containing bound sulfane sulfur have been regarded not only as H_2_S storage form but also as important molecules in redox regulation.

Hydrogen sulfide (H_2_S) was recognized as a gasotransmitter about thirty years ago. It is synthesized endogenously in mammalian cells from L-cysteine (Cys-SH) in reactions catalyzed by cystathionine β-synthase (CBS), cystathionine γ-lyase (CSE), and 3-mercaptopyruvate sulfurtransferase (MST). Synthesis of H_2_S from D-cysteine was also described in peroxisomes in reactions catalyzed by D-amino acid oxidase (DAO) and MST [21]. Another important source of H_2_S is the human microbiome with sulfate-reducing bacteria (SRB) able to reduce sulfate to H_2_S non-enzymatically [22,23]. H_2_S as a signaling molecule plays its physiological role only at relatively low concentrations, whereas in higher concentrations it is toxic due to an inhibition of cytochrome oxidase and impairment of the cell respiration [24,25]. H_2_S bioavailability is regulated through its conversion into different forms of sulfane sulfur or by its efficient catabolism. Two main pools of H_2_S storage, which can release free H_2_S under specific conditions, comprise bound sulfane sulfur and acid-labile sulfur. Bound sulfane sulfurs mainly include persulfides (RSSH), polysulfides (RS_n_R, n > 2), and inorganic polysulfides (H_2_S_n_), whereas acid-labile sulfur consists of iron-sulfur clusters contained in iron-sulfur proteins (Figure 4). The catabolism of H_2_S takes place in the mitochondria via the “sulfide oxidizing pathway”, with thiosulfate and sulfate as the end products of this pathway. The mitochondrial oxidation of H_2_S is catalyzed by sulfide quinone oxidoreductase (SQR), persulfide dioxygenase (ETHE1), and rhodanese (TST) [26]. Currently, it is known that persulfides and polysulfides are largely responsible for the biological actions attributed to H_2_S [27]. These reactive sulfur species are synthesized by the same enzymes as those engaged in the formation of H_2_S. During the mitochondrial H_2_S oxidation pathway, persulfides (e.g., sulfide quinone oxidoreductase persulfide, SQRSSH, and glutathione persulfide, GSSH) are formed as the intermediates [26]. Moreover, these two forms of reactive sulfur can easily merge into each other (Figure 4). Persulfides and polysulfides, unlike H_2_S itself, can react with protein Cys groups leading to persulfidation. This process is a kind of reversible covalent modification of proteins that can change their function [28]. Therefore, when considering the physiological role of H_2_S and its many pathological aspects including COVID-19, it should also be taken into account that some of these effects are connected with persulfides and polysulfides. During the mitochondrial H_2_S oxidation pathway, persulfides (e.g., sulfide quinone oxidoreductase persulfide SQRSSH and glutathione persulfide GSSH) are formed as the intermediates [26] (Figure 4).

It has been well-documented that H_2_S plays an important physiological role in the nervous system [29], in the circulatory system [30], and in renal physiology [31,32]. H_2_S has been also recognized as a mediator and therapeutic agent in diabetes [33,34] and inflammation [35]. Recent studies that focused on COVID-19 drew attention to the potential modulatory role of H_2_S in this viral respiratory disease.

The influence of H_2_S on ACE2 has already been documented in an artery mouse model and cardiomyocytes [36]; however, the effect of H_2_S on ACE2 in pulmonary tissue has, so far, not been sufficiently investigated and needs to be validated. Only one study tested the in vitro effects of H_2_S donors (NaHS and GYY4137) on ACE2 and TMPRSS2 expression in human upper- and lower-airway epithelial cells. The authors showed that H_2_S significantly reduced the expression of TMPRSS2 but that ACE2 mRNA expression was not modulated by the used H_2_S donors, neither in bronchial nor in pulmonary cells [37]. In a prostate cell model, it was found that TMPRSS2 transcription could be inhibited by H_2_S [38]. Hence, it can be hypothesized that in numerous pathological conditions associated with the reduced synthesis of endogenous H_2_S (e.g., diabetes, chronic kidney disease, cardiovascular diseases) TMPRSS2 is overexpressed, which facilitates the entry of SARS-CoV-2 into the host cells. It can be concluded that H_2_S can block the entry of SARS-CoV-2 into the host cells by a down-regulation of TMPRSS2 expression and probably by an inhibition of ACE2 activity.

The possibility of viral entry interruption is not the only option for the anti-SARS-CoV-2 action of H_2_S. There are some evidences demonstrating that H_2_S or other reactive sulfur compounds can inhibit many pathogenic RNA viruses [8,39,40,41]. In the case of respiratory syncytial virus (RSV), it has been observed in an in vitro study that H_2_S diminished the viral replication in A549 cells [41]. Moreover, the inhibitory effect of an H_2_S donor on RSV replication was also confirmed in an in vivo study using a mouse model [42]. Interestingly, it has been documented that infection with RSV inhibits CSE expression and induces expression of SQR, thereby contributing to the reduction in H_2_S in airway epithelial cells A549. On the other hand, these authors observed increased viral replication in cells treated with a specific CSE inhibitor [41]. Moreover, another study demonstrated that the CSE gene knockout mice developed enhanced RSV-induced lung damage and viral replication compared to the control mice [43]. The effect of H_2_S has also been studied in other RNA virus models. For example, in an in vitro model of influenza infection, the commonly used H_2_S donor, GYY4137, decreased the expression of influenza viral mRNA [39]. According to these findings, it seems that H_2_S donors could be also effective in inhibiting the replication of SARS-CoV-2, however, there is currently no direct evidence.

Serious cases of SARS-CoV-2 have been shown to be associated with the pro-inflammatory response and cytokine storm [44,45]. High levels of pro-inflammatory cytokines, including IL-1β, IL-6, IL-8, and TNF have been found in SARS-CoV-2 patients [46]. IL-6 has been considered the main and most relevant parameter in predicting the most severe course of respiratory failure, lung injury, and death in COVID-19 [47]. It has been documented in earlier studies that H_2_S is an effective down-regulator of IL-6 [48,49]. Moreover, recent studies also confirmed that H_2_S or its naturally occurring donors including diallyl disulfide or sulforaphane significantly reduced IL-6 and IL-8 release, inhibited TNF, and increased the anti-inflammatory IL-10 [50,51,52]. Recently, Renieris et al. evaluated the levels of IL-6, CRP, and TNF as well as the level of H_2_S in the serum of patients with COVID-19 [53]. Interestingly, their study revealed a correlation between the H_2_S level and the severity of disease progression, final outcome, and cytokine production. A significantly higher serum level of H_2_S was detected on the 1st and 7th day after admission to hospital in survivors, whereas mortality was significantly greater among patients with decreased H_2_S levels. Moreover, serum H_2_S on day 1 was negatively correlated with the level of IL-6 and CRP. The authors concluded their study by proposing H_2_S as a potential marker for the severity and final outcome of pneumonia induced by SARS-CoV-2 [53].

One of the well-established mechanisms of the anti-inflammatory action of H_2_S is related to the inhibition of NF_Κ_B activity. H_2_S blocks NF_Κ_B activation through persulfidation of IκB bound to NF_Κ_B. In this way, H_2_S prevents the translocation of NF_Κ_B into the nucleus [54]. This mechanism seems to be important as an anti-inflammatory action of H_2_S in COVID-19 therapy since the excessive NF_Κ_B activation is involved in the lung inflammatory process induced by SARS-CoV-2. Moreover, it has been documented that the SARS-CoV-2 spike protein is associated with increased degradation of IκB, leading to the NF_Κ_B signaling pathway activation [55,56].

It has been found that physiological concentrations of H_2_S inhibit the activity of the NLRP3 inflammasome and reduce the production of pro-inflammatory cytokines in vitro and in vivo [57,58]. On the other hand, a decreased level of H_2_S has been reported in the plasma of patients with COVID-19 [53,59], which suggests increased activity of NLRP3 and an aggravation of inflammation.

Viral diseases, including COVID-19, are accompanied usually by an overproduction of thick, difficultto-remove mucus. Mucins, the major mucus proteins, are rich in Cys-SH and can form massive aggregates, which are stabilized by intra- and intermolecular disulfide bridges [60]. It has been documented that H_2_S is able to modulate mucolytic activity and make the mucus less viscous. This appears to result from the interactions of H_2_S with the disulfide bonds of mucins, leading to reductions in the latter, which makes the mucus less viscous [61]. Moreover, exogenous H_2_S shows an antiabsorptive effect on the electrolyte-absorbing pulmonary epithelia [62]. This action of H_2_S leads to an increase in mucociliary clearance and makes the elimination of foreign microorganisms more effective [61].

More recently, Dominic et al. assessed the relationship between the availability of various biological pools of reactive sulfur including free H_2_S, bound sulfane sulfur, and acid-labile sulfur, and the NO level in patients with COVID-19 [63]. The obtained results clearly demonstrated a significantly reduced level of total and free sulfides and acid-labile sulfur. Among various studied sulfide metabolites, only bound sulfane sulfur remained unaffected by COVID-19. Moreover, the levels of total NO, free nitrite, and S-nitrosothiol metabolites have been found to be diminished in the serum of COVID-19 patients [63]. These results unquestionably indicate that COVID-19 is a pathology that is related to disturbances in the RSS homeostasis. Moreover, both above-cited papers suggest that free H_2_S or its total and acid-labile forms can be useful as prognostic biomarkers in a course of COVID-19.

## 3. NAC as a Drug for COVID-19 Treatment

N-acetylcysteine (NAC) is the N-acetyl derivative of the amino acid Cys. NAC easily penetrates cells where it is deacetylated to yield Cys, thereby promoting GSH synthesis. The thiol group confers antioxidant effects and is able to reduce ROS. NAC has aroused scientific interest for decades due to its important biochemical and pharmacological properties. It is useful as a mucolytic agent for the treatment of chronic bronchitis and other pulmonary diseases complicated by the production of viscous mucus [64]. It is also used as an antidote to paracetamol (acetaminophen) poisoning [65]. Although the therapeutic properties of NAC have been known for over half a century, subsequent scientific reports indicate an increasingly wider spectrum of its pharmacological activity. More recent studies have investigated the potential use of NAC in psychiatric and neurological disorders including Alzheimer’s and Parkinson’s diseases, schizophrenia, depression, bipolar disorder [66,67,68,69], and drug use disorder [70]. Moreover, the available data suggest that NAC may be beneficial in preventing the cognitive decline associated with both acute physiological insults and dementia-related disorders [71,72]. NAC has been also used to treat acute liver failure [73], spermatogenesis disorders [74], and dermatological diseases [75]. Furthermore, NAC exerts antiviral effects. In studies conducted in H5N1-infected lung epithelial (A549) cells, Geiler et al. showed that NAC inhibited the replication of seasonal human influenza A viruses and decreased the production of pro-inflammatory molecules. In the authors’ opinion, the antiviral and anti-inflammatory mechanisms of NAC action are associated with the inhibition of the activation of oxidant-sensitive pathways, including transcription factor NFκB and mitogen-activated protein kinase p38 [76]. It has also been demonstrated that the daily administration of NAC at 600 mg to postmenopausal women strengthened their immune defenses thereby decreasing the probability of immune system-related diseases in aging, including infections. The effects of NAC were monitored by measuring several lymphocyte functions (adherence, chemotaxis, proliferation, and natural killer activity), neutrophil functions (adherence, chemotaxis, phagocytosis, and superoxide), as well as cytokine levels, such as IL-2, IL-8, and TNF-α [77]. Interestingly, one study described the use of NAC in a one-month-old full-term male (4.5 kg) who was diagnosed with gastric lactobezoar, which is an aggregation of mucus and undigested milk and can obstruct any part of the digestive tract. This illness was successfully treated with a 10 mg/kg/dose of NAC that was given every 6 h for a total of 4 days [78]. Taking into account all the above-mentioned activities of NAC, it is not surprising that in looking for effective drugs to treat severe cases of COVID-19, attention was drawn to NAC.

One of the first clinical studies revealed a therapeutic blockade of inflammation in severe COVID-19 infection by NAC. The drug was administrated intravenously to COVID-19-infected respirator-dependent patients and a clinical improvement was observed in all patients [79]. Moreover, in this study, a severe case of COVID-19 infection in a glucose 6-phosphate dehydrogenase-deficient patient was described. This deficiency facilitates infection due to GSH depletion and predisposes erythrocytes to hemolysis. Treatment of this patient with hydroxychloroquine together with an intravenous administration of NAC allowed his removal from the respirator reduced his CRP, and blocked the hemolysis [79]. This study, however promising, is unreliable because it was conducted in a very small group of nine patients and only a few biochemical parameters were measured. A recent two-center retrospective cohort study provides much more information related to the effectiveness of NAC in the treatment of COVID-19 [80]. This study included 82 patients hospitalized with moderate or severe COVID-19 pneumonia and half of them, apart from standard care, received additional NAC at a dose of 1200 mg orally for 14 days. The second half of the patients received standard care. The obtained results revealed that the treatment with NAC slowed down the progression to severe respiratory failure compared to the control group. A lower mortality rate was observed in patients treated with NAC compared to the controls. These results confirm the hypothesis about the potential of NAC and its use as adjunctive therapy in COVID-19. The great advantages of NAC include the low cost of therapy, and the high tolerability and safety of NAC, even when high doses are used for a relatively long time.

NAC is offered as a drug to treat COVID-19 due to its well-documented anti-inflammatory and antiviral activity [81]. It has been demonstrated that NAC inhibits the NFκB pathway and reduces the replication of human influenza viruses in human lung epithelial cells. Moreover, NAC exhibits anti-inflammatory properties reducing the production of proinflammatory cytokines IL-6 and IL-8 [76,82]. Some papers suggest that the oral administration of NAC significantly decreased the frequency and severity of influenza and other viral diseases and could reduce the incidence of pneumonia [83] as well as improve oxygenation and reduce the need for ventilation support in acute lung injuries [84]. Some studies performed in oxygen–glucose-deprived rat cardiomyocyte H9C2 that was used as the cellular model for myocardial infarction, revealed that NAC suppressed the activity of NLRP3 and the activation of GSDMD and also reduced the NFκB [85].

**Figure 5:**
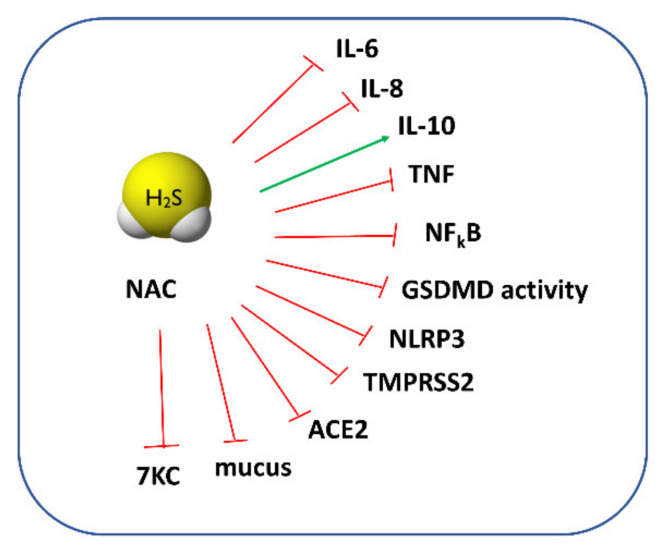
Potential protective role of H_2_S and NAC against COVID-19. H_2_S and NAC exert anti-inflammatory action through inhibition of NLRP3 inflammasome activity, GSDMD activity, reduction of proinflammatory cytokine production (IL-6 and IL-8), an increase in anti-inflammatory cytokine IL-10. The activity of tumor necrosis factor (TNF) and transcription factor NF_k_B can also be inhibited by H_2_S and NAC. H_2_S and NAC can block the entry of SARS-CoV-2 into host cells by suppressing the transmembrane serine protease 2 (TMPRSS2) activity and the receptor angiotensin-converting enzyme 2 (ACE2) activity. Moreover, NAC and H_2_S can modulate mucolytic activity and make the mucus less viscous and reduce the level of 7-ketocholesterol (7KC).

For a long time, NAC has been regarded mainly as a mucolytic agent, powerful antioxidant, and GSH precursor. However, when we carefully review the available papers regarding this topic, some of them do not support this activity. For example, a study by Ehre et al. has found that NAC is ineffective in altering sputum biophysical properties due to its low reducing activity [86]. It has also been thought that NAC can protect cells against oxidative stress through direct-scavenging ROS. However, as recently analyzed by Pedre et al., the rate constants of this reaction are rather slow, suggesting that other endogenous thiols (i.e., GSH) may be more efficient in ROS scavenging [87]. An increase in the GSH concentration after NAC administration is also questionable since some reports have not found changes in the GSH level or have shown that NAC exerts a beneficial effect even when the synthesis of GSH was blocked [88]. Given the documented beneficial effects of NAC and no explanation of its cytoprotective mechanisms, it seems that it can be converted inside cells to other species possessing stronger antioxidant properties. A study by Ezerina et al. found that NAC functions as an antioxidant by triggering the production of intracellular H_2_S and other sulfane sulfur-containing compounds [89]. In this aspect, NAC could serve as a precursor of other reactive sulfur species, such as H_2_S or sulfane sulfur [90], and its action could be particularly beneficial in patients with severe COVID-19 since, as mentioned above, a low H_2_S concentration was detected in the serum of these patients. The proposed protective action of H_2_S and NAC against COVID-19 is presented in Figure 5. It seems that NAC derivatives, such as NAC polysulfide [91], superoxide-responsive persulfide donor (SOPD-NAC) [92], and ester disulfide prodrug (EDP-NAC) [93], used as polysulfide or persulfide precursors can also be useful in the treatment of COVID-19. NAC persulfides are regarded as a powerful antioxidant and reductive species, and it could be expected that they will exert even greater anti-inflammatory and antiviral effects than NAC.

Interestingly, NAC also influences the metabolism of oxysterols, which are oxidized forms of cholesterol and often show greater biological activity than cholesterol itself. Oxysterols are characterized by their pleiotropic effects on various types of cells in the body and, therefore, often have opposite effects. Unlike cholesterol, they can cross the blood–brain barrier and affect the functioning of the nervous system. Many studies have shown that oxidized forms of cholesterol can regulate the activity of the cells of the immune system and, thus, can have an impact on the immune response in the course of viral diseases, including COVID-19 [94]. The importance of oxysterols in the course of infectious diseases is additionally emphasized by the fact that in the acute phase of COVID-19, the blood level of the antiviral 27-hydroxycholesterol significantly decreases, whereas the levels of 7β-hydroxycholesterol and 7-ketocholesterol (7KC) increase [95]. However, it is known that 7KC can contribute to the pathophysiology of COVID-19 due to its pro-oxidant and pro-inflammatory properties as well as its ability to promote cell death, which, combined with disturbances such as high BMI, diabetes, dyslipidemia, and cardiovascular disease, increases the risk of severe COVID-19 [96]. Hence, many authors postulate the use of statins in patients with severe COVID-19 to reduce the level of cholesterol and thus oxysterols, by inhibiting the 3-hydroxy-3-methylglutaryl-CoA reductase, which is the key enzyme for the synthesis of cholesterol [96,97]. Other compounds that could lower the level of “bad oxysterols”, mainly 7KC and 7β-hydroxycholesterol, are also being sought to assist in the treatment of COVID-19 patients. Among such compounds, numerous natural compounds such as vitamins and antioxidants are mentioned [98].

Studies conducted on the human umbilical vein endothelial cells (HUVECs) showed that the 7β-hydroxycholesterol-stimulated production of ROS was inhibited in the presence of NAC [99]. In addition, Wang et al. revealed in in vitro studies that the increasing effect of 7KC on the level of superoxide radicals was eliminated by a 30 min pretreatment of cells with NAC [100]. A study by Lizard et al. demonstrated that the addition of NAC was able to impair the 7KC-induced apoptosis [101]. Taken together, NAC can exert an additional beneficial effect in the course of COVID-19 by lowering the level of 7KC that is increased in the plasma of patients with severe forms of COVID-19. It can be especially important in the case of elderly patients because the increased level of toxic 7KC is associated with many disabilities related to aging [102]. All aspects of the pharmacological action of NAC and H_2_S are presented in Figure 5.

## 4. Disulfiram in COVID-19 Treatment

Disulfiram (1-(diethylthiocarbamoyldisulfanyl)-N,N-diethyl-methanethioamide, DSF, also known by other names: tetraethylthiuram disulfide, antabuse) is a derivative of thiuram and a well-known inhibitor of aldehyde dehydrogenases (ALDHs) that catalyzes the oxidation of aldehydes to carboxylic acids. Among the 19 enzymes of the ALDH family, ALDH class-2 (ALDH2), a mitochondrial enzyme highly expressed in the liver, plays a major role in the acetaldehyde metabolism into nontoxic acetic acid [103]. By inhibiting ALDH2 activity, DSF causes ethyl alcohol intolerance due to poisoning with acetaldehyde, the concentration of which is high after ethanol consumption. For this reason, DSF has been a drug used in alcohol aversion therapy for over 70 years. It should be added that drinking alcohol during treatment with DSF not only makes one feel unwell but can also be life-threatening. From this point of view, the use of DSF as a treatment in patients with alcoholism is regarded by many people as an unethical therapy because it is connected with the risk of poisoning and even a loss of life.

On the other hand, recent studies indicate that DSF is able to inhibit other enzymes, by reacting—as in the case of ALDH—with important Cys residues, suggesting its pharmacological activity. For this reason, DSF has been studied as a possible treatment for cancer [104], parasitic infections [105], and latent human immunodeficiency virus HIV infection [106].

In 2018, it was shown that DSF might inhibit the papain-like protease (PLpro) of MERS-CoV and SARS-CoV. In the same study, it was also shown that DSF acted as an allosteric inhibitor of MERS-CoV PLpro but as a competitive (or mixed) inhibitor of SARS-CoV PLpro [107]. It is worth recalling that PLpro is an essential coronavirus enzyme that is required for processing viral polyproteins to generate a functional replicase complex and to enable viral spread. Currently, DSF is increasingly studied as a possible treatment for SARS-CoV-2 infection. Tamburin et al. explored whether patients treated with DSF for alcohol use disorder (AUD) had reduced COVID-19 and related symptoms. It was a multicenter observational retrospective study based on telephone interviews with patients aged > 18 with AUD living in Northern Italy (Lombardy, Veneto, Emilia Romagna, Piedmont, and Liguria regions), where the first COVID-19 peak was more severe in spring 2020. Admittedly, the authors found no significant difference in the incidences of laboratory-confirmed COVID-19, related hospitalization, or pneumonia, but the symptoms compatible with COVID-19 were significantly less common in DSF group [108]. In turn, Fillmore et al. investigated the potential effects of DSF on SARS-CoV-2 infection and disease severity in an observational study using a large database of clinical records from the national US Veterans Health Administration system. Statistical analysis of the obtained data revealed a reduced risk of SARS-CoV-2 infection with DSF use at a hazard ratio of 0.66 (34% lower risk, 95% confidence interval 24–43%). Moreover, there were no COVID-19-related deaths among the 188 SARS-CoV-2-positive patients treated with DSF, in contrast to the 5–6 statistically expected deaths based on the untreated population. These epidemiological data obtained by the authors suggest that DSF may contribute to the reduced incidence and severity of COVID-19 [109].

**Figure 6:**
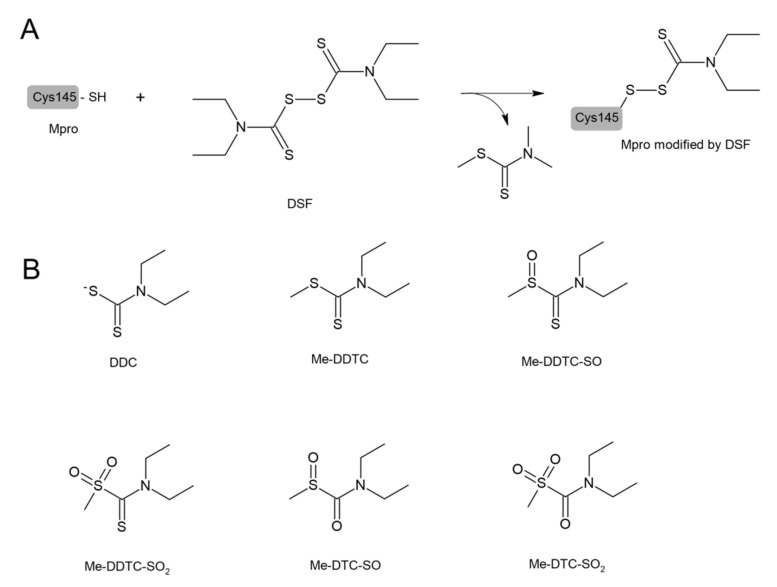
(**A**) A possible mechanism of modification of the catalytic Cys145 of SARS-CoV-2 Mpro by disulfiram (DSF) leading to the inhibition of Mpro activity. (**B**) The products of DSF metabolism that have the ability to inhibit SARS-CoV-2 include N,N-diethyldithiocarbamate (DDC); S-methyl N,N-diethyldithiocarbamoyl sulfide (MeDDTC); S-methyl-N,N-diethyldithiocarbamoyl sulfoxide (MeDDTC-SO); S-methyl-N,N-diethyldithiocarbamoyl sulfone (MeDDTC-SO2); S-methyl-N,N-diethylthiocarbamoyl sulfoxide (MeDTC-SO); and S-methyl-N,N-diethylthiocarbamoyl sulfone (MeDTC-SO2). The mechanism of the Mpro inhibition by these compounds is similar to that presented for DSF (for details see [112]).

DSF and other structurally diverse compounds have also been found to be the main protease (Mpro) inhibitors. It has been recently reported that DSF inhibited the SARS-CoV-2 Mpro with an IC50 value of 9.35 ± 0.18 μM that was assayed by fluorescence resonance energy transfer [110]. Ma et al. demonstrated that DSF was a promiscuous cysteine protease inhibitor that inhibited both the Mpro and PLpro of SARS-CoV-2 in the absence of dithiothreitol (DTT) and that this inhibition was abolished by the addition of reducing reagents (DTT or GSH) [19]. Lobo-Galo et al. have provided in silico data showing that the catalytic Cys145 of Mpro from coronavirus SARS-CoV-2 could be blocked and inactivated by DSF [97], which is presented in Figure 6. DSF in in vivo conditions was rapidly reduced to form N,N-diethyldithiocarbamate (DDC), which is a DSF monomer. Other metabolites of DSF include S-methyl N,N-diethyldithiocarbamoyl sulfide (MeDDTC); S-methyl-N,N-diethyldithiocarbamoyl sulfoxide (MeDDTC-SO), S-methyl-N,N-diethyldithiocarbamoyl sulfone (MeDDTC-SO2), S-methyl-N,N-diethylthiocarbamoyl sulfoxide (MeDTC-SO), and S-methyl-N,N-diethylthiocarbamoyl sulfone (MeDTC-SO2). It has been suggested that not only DSF but also the products of its metabolism, have the ability to inhibit SARS-CoV-2 virus replication by modification of the Cys145 thiol group, similar to DSF [111] (Figure 6).

It has also been found that DSF can affect GSDMD activity and pyroptosis. A recent study by Hu et al. revealed that DSF inhibited both pyroptosis and cytokine release in cells and lipopolysaccharide (LPS)-induced septic death in mice. It has also been shown that at nanomolar concentrations, DSF covalently modifies human/mouse Cys191/Cys192 in GSDMD leading to a blockade of the pore formation. According to the authors, the role of DSF in inhibiting GSDMD provides new therapeutic indications for repurposing this well-known drug to counteract inflammation, which contributes to many human diseases [113]. Moreover, Adrover et al. demonstrated that DSF under experimental conditions appeared to confer more benefits on lung pathology in SARS-CoV-2-infected hamsters than dexamethasone, widely used for COVID-19 treatment. The authors also showed that dexamethasone, but not DSF, significantly increased the viral load in the lungs when administered from day 1 post-infection [114]. It is worth adding that many authors warn against the unfavorable effects of corticoid therapies on respiratory viruses [115]. In our opinion, if further research reliably confirms that DSF is a more beneficial alternative to corticoids in the treatment of inflammation of the respiratory tract, it will be a real breakthrough in practical clinical medicine.

**Figure 7:**
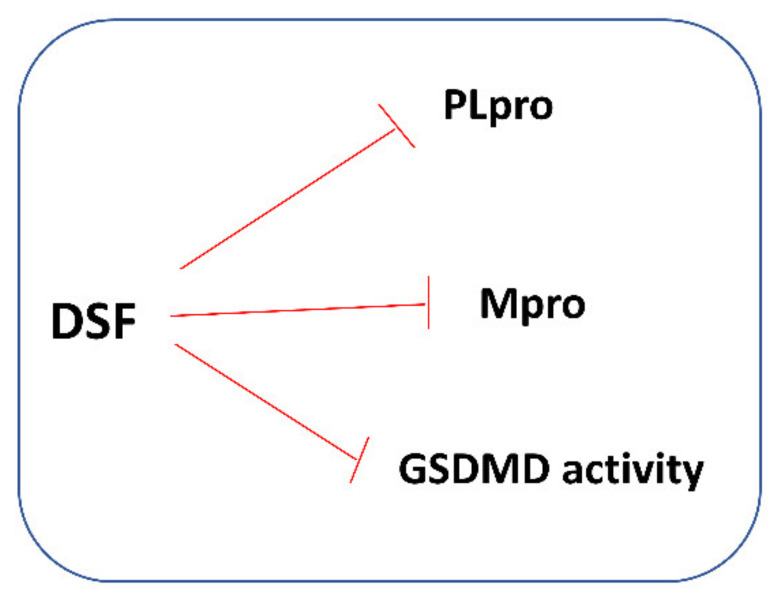
Some mechanisms of protective action of DSF against COVID-19. DSF inhibits the papain-like protease (PLpro) and main protease (Mpro) of SARS-CoV-2; both enzymes are essential to the replication and transcription of the virus. DSF can also affect GSDMD activity and in this way inhibit pyroptosis and cytokine release.

Thus, in light of the presented data, it seems appropriate to recommend testing DSF—an alcoholism-averting drug—for its potency to control SARS-CoV-2 infection, considering that it has been previously proposed as an antimicrobial and anti-SARS and MERS agent, safe to use even at higher doses, and with a low side-effect profile. Some mechanisms of the protective action of DSF against COVID-19 are presented in Figure 7.

## 5. Lipoic Acid (LA) and COVID-19

**Figure 8:**
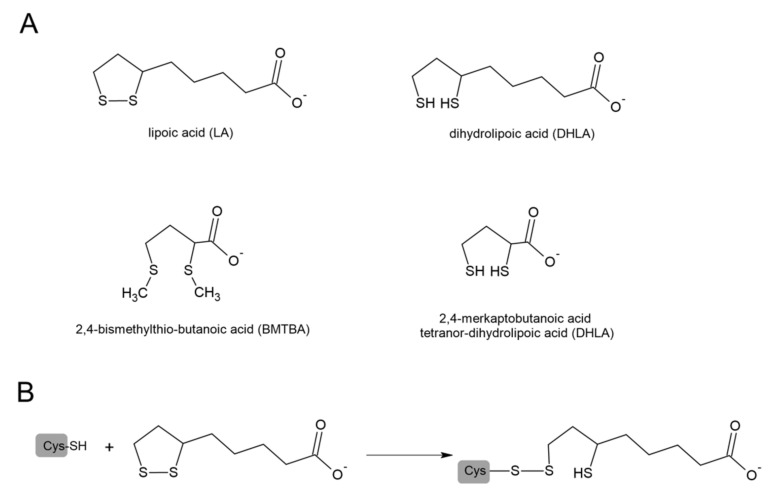
(**A**) Structures of lipoic acid (LA), dihydrolipoic acid (DHLA), and products of its biotransformation: 2,4─bismethylthio-butanoic acid (BMTBA) and tetranor-dihydrolipoic acid (tetranor-DHLA). (**B**) Modification of Cys residue by LA through thiol–disulfide exchange (modified from [126]).

Lipoic acid (1,2-dithiolane 3-pentanoic acid, LA) and its reduced form dihydrolipoic acid (DHLA) are naturally occurring sulfur compounds. In mammals, LA is synthesized in very small quantities in the liver and other tissues and is used as a coenzyme indispensable for the activity of multienzymatic complexes that play a key role in mitochondrial energy metabolism. In these enzymatic complexes, LA is linked by an amide bond to the ε-amino group of a lysine residue of the protein. A beneficial effect of LA was confirmed in cancer, diabetes, and neurodegenerative and cardiovascular disorders [116]. It has been suggested that the mechanisms of LA’s therapeutic action are based on the strong antioxidant properties of the LA/DHLA system. Looking at the potential redox values, we can see that DHLA can participate in reduction reactions, both neutralizing ROS and regenerating oxidized forms of other antioxidants, including oxidized glutathione (GSSG) [117,118]. Goraca and Skibska reported protective effects of early LA administration against LPS-induced oxidative stress in the rat lung [119]. Liu et al. demonstrated that LA also attenuated LPS-induced liver injury, which was evidenced by the determination of the plasma alanine and aspartate aminotransferases [120]. Some studies also suggest that not only LA but also products of its degradation show anti-inflammatory activity. The animal studies indicated that zymosan-induced peritonitis was significantly inhibited in groups receiving 2,4-bismethylthio-butanoic acid (BMTBA), which is the product of LA biotransformation. Moreover, the same study reported anti-inflammatory effects of BMTBA and a DHLA analog—tetranor-dihydrolipoic acid (tetranor-DHLA) in the carrageenan-induced hind paw edema models in mice [121]. The structures of LA and its derivatives are presented in Figure 8.

Antiviral effects of LA have also been reported. The study of Jariwalla et al. indicated that supplementation with LA might positively impact patients with HIV and acquired immune deficiency syndrome [122]. In turn, Berkson described the positive effects of LA administered together with silymarin and selenium in three patients with cirrhosis, portal hypertension, and esophageal varices secondary to chronic hepatitis C infection. The applied therapy eliminated the need for liver transplantation [123]. The antibacterial effect of LA has not been so clearly demonstrated, however, there are several studies showing its effectiveness in this area [124,125].

An increasing body of literature data indicates that LA may boost also human host defense against SARS-CoV-2 [127,128]. It is commonly known that the LA/DHLA system inhibits NF_k_B signaling with a decrease in pro-inflammatory cytokine secretion. Moreover, by preventing the oxidative depletion of tetrahydrobiopterin (BH4), an essential cofactor for the production of NO by the NO synthases (NOS), LA can restore the NOS activity resulting in an increase in NO bioavailability, and thus, it can improve the endothelial function [129]. It is also necessary to remember that the LA/DHLA system can directly scavenge the ROS or can restore the reduced form of GSH and other antioxidants, thus enhancing endogenous antioxidant protection. Moreover, LA stimulates GSH synthesis by increasing cellular Cys uptake and by activating the nuclear erythroid 2-related factor 2—antioxidant response element (Nrf2—ARE) signaling pathways. Nrf2 is a basic region leucine-zipper transcription factor that binds to the ARE, thereby regulating the expression of a large battery of genes involved in the cellular antioxidant and anti-inflammatory defense as well as mitochondrial protection [130]. Studies on the HepG2 cell line have shown that cadmium induced cell death by a depletion of GSH through the inactivation of Nrf2. On the other hand, when LA was added to cadmium-treated cells, Nrf2 reactivation and GSH regeneration were observed by elevating the Nrf2-downstream genes γ-glutamate-cysteine ligase (γ-GCL) and glutathione reductase (GR), both of which are key enzymes for GSH synthesis [131]. Moreover, as a cofactor of a few multienzymatic mitochondrial complexes catalyzing oxidative decarboxylation of α-ketoacids (pyruvate, α-ketoglutarate, branched-chain α-ketoacids, α-ketoadipate), LA/DHLA influences the supply of reducing equivalents (NAD^+^/NADH). This complex analysis could open a new therapeutic perspective for LA in COVID-19 infection.

A study in 17 patients critically ill with COVID-19 in Wuhan JinYinTan Hospital demonstrated that LA treatment improved the 30 day survival rate of those patients and slowed down the increase in the Sequential Organ Failure Assessment (SOFA) score, however, both parameters did not reach statistical significance due to the limited number of patients. According to the authors, the efficiency of LA in those patients was related to the activity of LA as an antioxidant and anti-inflammatory agent [132]. Incidentally, SOFA is a validated scoring system used to predict mortality in intensive care unit (ICU) patients. The higher the SOFA score, the higher the likelihood of a patient’s death [20]. Hummel et al. indicated that LA may be helpful in patients with post-upper respiratory tract infection olfactory loss, which could fill a therapeutic void. According to the authors, the possible mechanisms of action include the release of nerve growth factors and antioxidant effects, both of which may be helpful in the regeneration of olfactory receptor neurons [133]. Based on the literature, Sayıner et al. hypothesized that LA could decrease the ACE2 activity during infection with SARS-CoV-2 and could reduce the NADPH oxidase activity leading to a suppression of the increase in cytokine expression [134].

Using the model of LPS-stimulated human epithelial lung cells that mimics the pathogen-associated molecular pattern and reproduces the cell signaling pathways in cytokine storm syndrome, it was shown that a combination of 50 µM LA and 5 µM palmitoylethanolamide (PEA) could reduce ROS and NO levels modulating the major cytokines involved in COVID-19 infection [135]. The best outcome was observed when LA and PEA were administered after LPS, thus reinforcing the hypothesis that LA combined with PEA is able to modulate the key point of cytokine storm syndrome. In the authors’ opinion, these results for the first time provide support to the conclusion that the combination of LA with PEA may represent a novel intervention strategy to counteract inflammatory damage related to COVID-19 by restoring the cascade activation of the immune response and by acting as a powerful antioxidant [135].

Data indicate that LA can activate ATP-dependent K^+^ channels (Na^+^,K^+^-ATPase) [136,137]. Cure and Cure noted that LA, through activation of these channels, could increase the intracellular pH (Na^+^,K^+^-ATPase pumps 2K^+^ into the cell and 3Na^+^ outside the cell). In this way, LA was able to decrease the risk of the virus’ entry into the cell and increase the human host’s defense against SARS-CoV-2 [138]. In addition, the authors observed that the use of LA with insulin in patients with diabetes showed a synergistic effect against SARS-CoV-2, therefore, LA treatment will be beneficial against COVID-19 in patients with diabetes [138]. McCarty et al. in their review, highlight the importance of suppressing NLRP3 inflammasome activation in the management of COVID-19 [139]. It has been reported that H_2_S inhibits NLRP3 inflammasome activity [57], which means that compounds that support H_2_S synthesis or are its precursors may aid this effect. It should be noted that Mikami et al. demonstrated that 3-mercaptopyruvate sulfurtransferase (MST) produced H_2_S from thiosulfate only in the presence of such factors as DTT or DHLA, but in the presence of DHLA, the production of H_2_S was greater than in the presence of DTT [140]. Studies by other authors also show that the biological actions of LA may be associated with sulfane sulfur and H_2_S metabolism [141,142,143]. Some authors suggested that LA could possibly participate in the thiol–disulfide exchange with critical redox-sensitive thiol groups according to the reaction presented in Figure 8B [126]. This process is analogous to the process of modification of Mpro by the above-mentioned DSF and its metabolites.

**Figure 9:**
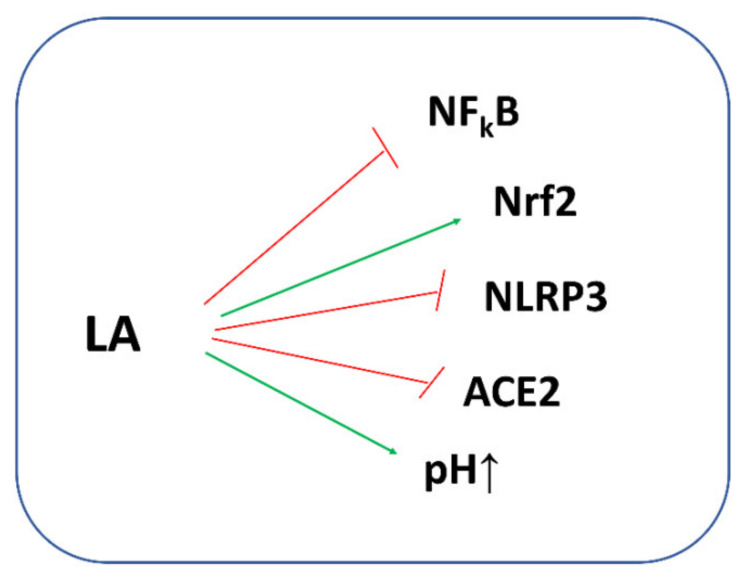
Biological potential of LA in the fight against COVID-19. LA exerts anti-inflammatory action via inhibiting the activity of NF_k_B and NLRP3 inflammasomes and activating the nuclear erythroid 2-related factor 2 (Nrf2). LA decreases the risk of SARS-CoV-2 entry into the cell by increasing the intracellular pH. Moreover, LA may decrease the activity of the angiotensin-converting enzyme 2 (ACE2) receptor.

The biological potential of LA against COVID-19 is presented in Figure 9.

## 6. Glutathione (GSH) in COVID-19

Glutathione is a ubiquitous tripeptide composed of glutamate (Glu), cysteine (Cys), and glycine (Gly) containing an unusual γ-peptide bond between Glu and Cys. GSH is synthesized in two ATP-dependent reactions catalyzed by glutamate-cysteine ligase (GCL) and glutathione synthase (GS). GSH fulfills its biological role in reduced, thiol form, however, it can also exist in the oxidized form of glutathione disulfide (GSSG). The level of intracellular GSH in the millimolar concentration range is balanced by its synthesis, regeneration from the GSSG, and extracellular GSH uptake catalyzed by γ-glutamyl transferase (γ-GT) and dipeptidase (DP). GSH is regarded as the main low molecular-weight antioxidant agent, which can scavenge free radicals directly or as a coenzyme for glutathione peroxidase (GPx). As a result of these reactions, GSH is oxidized to GSSG and then the reduced form of glutathione can be restored in the reaction catalyzed by the NADPH-dependent glutathione reductase (GR). GSH as a reductant fulfills an important role in protecting protein-SH residues from oxidation; it also takes part in the reduction of protein mixed disulfides (PSSG). Furthermore, GSH is involved in the detoxification of xenobiotics catalyzed by glutathione S-transferase (GST). It is believed that GSH plays a central role in the control of many processes, such as immune response, antiviral defense, detoxification, and protein folding [144].

Different risk factors including age, hypertension, heart disease, diabetes, obesity and other metabolic disorders, and smoking have been regarded as predisposing people to a severe course of COVID-19 and high mortality. In all these states, a decrease in the level of GSH was observed [145,146,147,148,149,150]. A depletion of GSH leads not only to a loss of its protective role in the organs but also to an impairment of the immune system function, especially the T lymphocytes and macrophages. This fact may be associated with the high incidence of secondary infection in patients with COVID-19 [151]. It has also been shown that GSH exerts antiviral effects against some viruses including influenza, dengue virus, and herpes simplex virus [111,152,153]. As suggested by Khanfar and Qaroot in their paper, due to the key role of GSH in antioxidant defense and in supporting the immune system, this tripeptide may be at the core of COVID-19 pathophysiology [151].

It has been found that different types of viruses, including influenza, deplete cellular GSH and promote a pro-oxidant environment in the infected cells [154]. Bartolini et al. studied the effect of viral infection induced by SARS-CoV-2 in Vero E6 cells, regarded as an in vitro model of COVID-19 infection, on GSH homeostasis. This study revealed that SARS-CoV-2 markedly decreased the cellular level of GSH and other thiols. The authors explained this effect mostly by a reduced capability of the infected cells to sustain the GSH synthesis due to the limited response of cells to NAC being a precursor of Cys regarded as a rate-limiting factor in GSH synthesis. An increased level of GSSG and the protein glutathionylation (PSSG) was also observed in Vero E6 cells infected by SARS-CoV-2 compared to control cells [155].

Moreover, studies performed in patients with COVID-19 confirmed that SARS-CoV-2 significantly affected GSH homeostasis. Kumar et al. measured the concentrations of GSH and GSSG as well as the concentration of an oxidative stress marker and oxidant damage marker in 60 adult patients hospitalized with COVID-19. They observed a severe GSH deficiency, increased oxidative stress, and elevated oxidative damage in all studied patients when compared to uninfected controls [29]. Moreover, patients were divided into age groups and the authors reported that elevated oxidative damage and depletion of GSH worsened with advancing age; however, the GSH deficiency was also present in young COVID-19 patients. The authors suggested that supplementation with NAC and Gly might afford effective, powerful cellular protection from oxidative stress and the depletion of GSH in COVID-19 patients [29]. NAC is a commonly known precursor of Cys regarded as the rate-limiting factor in GSH synthesis, whereas Gly is an important metabolite in many reactions and also the substrate needed for GSH synthesis. Some clinical trials have shown that supplementation with a combination of NAC and Gly successfully lowers oxidative stress, raises GSH levels, and importantly, does not trigger reductive stress [156,157].

One paper reported the efficacy of glutathione therapy in relieving dyspnea associated with COVID-19 pneumonia. It is a case study of two patients with dyspnea secondary to COVID-19 pneumonia that were administered a high dose (2000 mg per day) of GSH orally or intravenously. The authors noted an alleviation of dyspnea within 1 h of GSH use, and repeated administration of GSH was effective in further relieving respiratory symptoms [158]. It is the only paper reporting a successful clinical action of GSH in respiratory symptoms during COVID-19; unfortunately, it is limited to only two cases. Furthermore, it should be pointed out that in this study GSH treatment was accompanied by the administration of ascorbic acid and other sulfur compounds with powerful biological potential, i.e., NAC and LA. It can be concluded that the observed effect of dyspnea relief is a combined effect of all used treatments. The common mechanisms of the anti-inflammatory action of the used compounds, including GSH, LA, and NAC, involve the inhibition of NFκB activation.

GSH, similar to the above-discussed NAC, can also protect cells against 7KC-induced damage [101]. It is not surprising since NAC is regarded as a GSH precursor. Moreover, there is a lot of evidence that antioxidants, including endogenous and synthetic molecules as well as natural polyphenols, have the potential to decrease the level of 7KC, a major cholesterol oxidation product that is increased in patients with age-related diseases [98,159]. It can also be expected that the administration of GSH in severe COVID-19 cases would be beneficial for patients in the context of 7KC as adjuvant therapy or after the severe phase of infection has passed to prevent complications associated with a viral infection, especially in elderly patients.

## 7. Erdosteine and COVID-19

**Figure 10:**
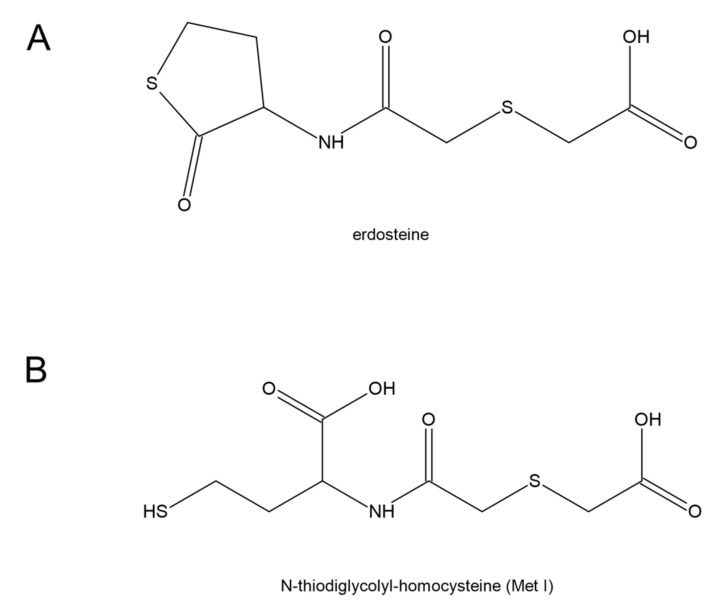
Structures of (**A**) erdosteine [N-(carboxymethylthioacetyl)-homocysteine thiolactone] and (**B**) its biologically active metabolite, N-thiodiglycolyl-homocysteine (Met I).

Erdosteine [N-(carboxymethylthioacetyl)-homocysteine thiolactone] is characterized by the presence of a carboxylic acid group and two sulfur atoms. It is metabolized in the liver to the biologically active metabolite, N-thiodiglycolyl-homocysteine (Met I) (Figure 10). Erdosteine is classified as a mucolytic agent [160], however, its much wider pharmacological properties have been suggested [161]. Erdosteine and Met I exert antibacterial effects by affecting the integrity of the tracheobronchial mucins and pilins. It is connected with a reduction in the disulfide bonds present in mucins and pilins by the sulfhydryl group of Met I. It has been proven that the combination of chosen doses of Met I and suitable inhibitory concentrations of ciprofloxacin potentiated the inhibition of *Staphylococcus aureus* and *Escherichia coli* adhesiveness to human mucosal cells in comparison with ciprofloxacin alone [162]. Moreover, experimental studies have documented that erdosteine prevents or reduces lung tissue damage induced by oxidative stress and, in particular, that Met 1 also regulates reactive oxygen species production [163,164]. The meta-analysis performed by Cazzola et al. has shown that erdosteine is able to improve the clinical score of patients with chronic bronchitis and chronic obstructive pulmonary disease (COPD) [165]. These data also suggest that erdosteine can lengthen the time to the first COPD exacerbation and reduce the risk of hospitalization from COPD.

In October 2020, Recipharm AB, a pharmaceutical industry Contract Development and Manufacturing Organization based in Stockholm, issued a press release to state that erdosteine had been positively tested as a part of COVID-19 treatment [166]. The clinical study involved 20 patients affected by COVID-19 with severe respiratory failure, hospitalized in one of the major COVID-19 treatment centers of Milan in Lombardy, an Italian region heavily affected by SARS-CoV-2. The study indicated that patients taking erdosteine after hospital discharge showed significant improvements in health-related quality of life parameters (HRQoL) and dyspnoea. As the authors point out, this study is one of the first to report HRQoL details in patients with COVID-19. The full study outcomes have been described in detail and published [167]. Santus et al. also observed that patients discharged from hospital after COVID-19-associated pneumonia often experienced persistent symptoms (e.g., dyspnea, cough, fatigue), which affected their quality of life [167]. The authors performed a single-center open-label study to assess the impact of the oral erdosteine (300 mg twice daily) for 30 days in 38 patients discharged from hospital after COVID-19-associated pneumonia who had persistent dyspnea. The patients completed a questionnaire and the modified Medical Research Council dyspnea scale at the time of discharge—day 0—and on day 30. The obtained results indicated that both scores improved significantly in the treatment group between days 0 and 30, whereas there were no significant changes in the control group. Moreover, on day 30, significantly more patients in the treatment group than in the control group achieved clinically important changes in HRQoL and symptoms [167].

Erdosteine has a unique anti-inflammatory profile among mucolytic drugs. It has been shown to reduce the production of isoprostane in the respiratory tract and to lower the plasma concentration of the C-reactive protein (CRP) [168,169]. As mentioned above, Met I, an active metabolite of erdosteine, having the -SH group, is capable of opening disulfide bonds, including those of pilin, a protein of bacterial fimbriae. This induces stereochemical conformational changes that interfere with the binding of bacterial adhesins (fimbriae) to receptors on eukaryotic cells. Thus, erdosteine inhibits biofilm formation and causes biofilm disruption, thereby improving the efficacy of antibiotic therapy [161,170,171]. This phenomenon is relevant to every situation in pneumonia or bronchitis. In COVID-19 patients, it is extremely important. It should be noted that mucolytic drugs are used quite commonly in COVID-19 patients. Erdosteine is one of the most advanced mucolytics on the market. It has been shown to reduce the number of recurrences of respiratory tract infections treated with antibiotics in children [172]. Moreover, erdosteine is a prodrug, has practically no side effects, shows no therapeutic interactions, and its efficacy was confirmed by adequate clinical trials in people of a wide age range [161].

## 8. Ergothioneine and Its Potential in COVID-19 Treatment

**Figure 11:**
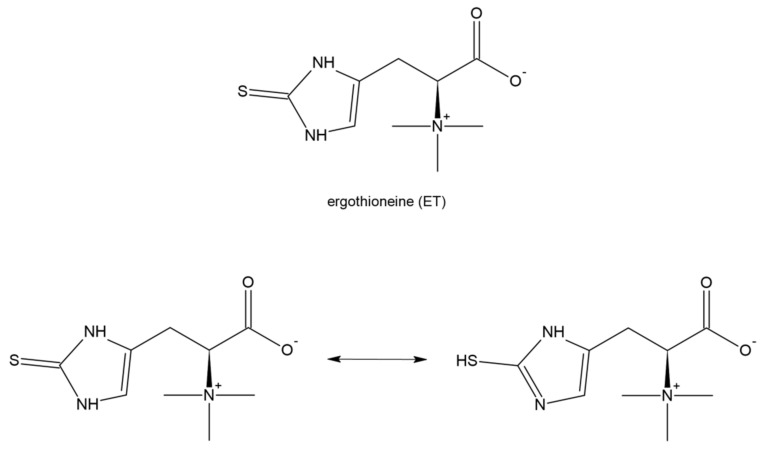
Structure of ergothioneine (ET), namely 2─mercapto-histidine-trimethylbetaine. ET exists in two tautomeric forms: thione and thiol, however, the thione form is prevailing.

Ergothioneine (2-mercaptohistidinetrimethylbetaine; ET) is a naturally occurring dietary amino acid with a sulfur atom on the imidazole ring (Figure 11). It is a thiol/thione molecule synthesized only by some fungi and bacteria. The tautomeric equilibrium favors the thione form. Dietary ET in animals (including humans) is absorbed using an intestinal transporter, OCTN1 (SLC22A4), that is, the first member of the human Novel Organic Cation Transporters small subfamily, which is a part of the larger SLC22 family [173,174]. ET is easily absorbed following oral consumption and is accumulated in many tissues including the liver, myocardium, and kidney which can suggest its important physiological role [175]. ET has the ability to scavenge reactive oxygen species, especially during increased oxidative stress, and decreased levels of ET have been observed in some diseases [176,177,178]. Moreover, several lines of evidence based on in vitro and in vivo studies show that ET produces anti-inflammatory action [179], protects against ischemia and reperfusion injury [180], and mitigates damage and fibrosis of the lung [181] and liver [182]. It has been reported that plasma concentrations of ET decrease with age [183] and on the other hand a low ET plasma level predisposes to an increased risk of cardiometabolic disease and enhanced mortality [184].

Recently, the effects of ET were studied in human brain endothelial cell lines treated with 7KC, the cholesterol oxidation product [185]. Endothelial cells are exposed to high levels of 7KC in patients with cardiovascular disease, diabetes, and severe COVID-19. 7KC induces a loss of cell viability and increases apoptosis or necrosis of the endothelial cells. The results obtained by Koh et al. revealed the anti-inflammatory effects of ET and suggested that ET might be useful in the prevention of some neurovascular diseases. Moreover, the authors discussed the possibility of ET use in COVID-19-related neurological complications [185].

All these facts allow the supposition that ET may exert beneficial therapeutic effects in COVID-19 or may provide prophylactic support, especially for the elderly, to reduce the risk of severe course of this disease. Unfortunately, no studies have described the application of ET for the treatment of COVID-19, yet. Only one review of the multidirectional biological actions of ET discussed the possibility of the use of this naturally occurring amino acid as a therapeutic to reduce the severity and mortality associated with COVID-19 [186]. Since ROS plays an important role in COVID-19 progression, antioxidants may be helpful in the treatment and shortening of the duration of this disease. The authors argue that ET has a high bioavailability, is actively taken up into cells, and is preferentially accumulated in tissues, especially those at a high risk of ROS-induced damage. Moreover, in contrast to the powerful antioxidant ascorbic acid, ET does not enter pro-oxidative reactions with iron or copper. Furthermore, it has been demonstrated that fungal extracts with ET as the major active component inhibit some viral proteases suggesting the possible activity of ET in the inhibition of the binding or replication of SARS-CoV-2 [186,187]. Taking into account all the above-mentioned facts, it seems that ET is worth exploring as a potential medicine for administration in COVID-19 to reduce the severity of the disease.

## 9. Concluding Remarks

The sulfur-containing molecules presented in this paper have significant therapeutic potential and their beneficial actions in many different diseases have been documented. The present review describes the potential role of sulfur compounds in the protection against infection induced by SARS-CoV-2 and their suitability for the inhibition of virus replication. As most of the sulfur compounds discussed in this review, including H_2_S, NAC, LA, erdosteine, and ET possess well-documented anti-inflammatory and antioxidant properties, they can be useful in the suppression of pro-oxidative processes and inflammation accompanying a mild or severe course of the disease. Moreover, H_2_S and NAC influencing TMPRSS2 expression and ACE2 activity can protect host cells against SARS-CoV-2 infection and in this way inhibit virus fusion. These molecules also have multiple benefits in age-related diseases, such as cardiovascular diseases, diabetes, inflammation, and neurological diseases. Therefore, there is additional interest in the use of the presented reactive sulfur compounds, especially in elderly patients with COVID-19.

It seems that low levels of GSH and H_2_S in the plasma of patients with a severe course of COVID-19 could be among the major causes of the severe symptoms of the disease. Therefore, the low level of GSH and diminished concentration of H_2_S in the plasma of COVID-19 patients can be regarded as markers predisposing them to a severe course of the disease. On the other hand, it can be expected that patients with disturbed homeostasis of GSH and H_2_S could be treated with substances able to improve the homeostasis, help alleviate the symptoms of the disease, and protect against its severe course. In this aspect, clinically used NAC seems to be an ideal and safe candidate. NAC is a precursor of Cys, which is a rate-limiting factor in the GSH synthesis. On the other hand, Cys is a substrate for H_2_S synthesis. The clinical study described in this review confirmed that NAC exerted beneficial anti-inflammatory effects and could be used as adjunctive therapy in COVID-19. In our opinion, LA is the second candidate that could be used as a supportive therapy in COVID-19 since it is a clinically used drug with powerful antioxidant potential and is safe even at high doses, similar to NAC. Moreover, all other sulfur compounds or their precursors described in this review are worth studying in the context of their usefulness in the treatment of COVID-19 due to their biological activity connected with modulation of the inflammatory response and regulation of the host response to viral infections. However, in the case of all these compounds, additional data and studies are needed to confirm their effectiveness and safety before these molecules can be used in infected patients.

## Data Availability

Data contained in the article.

## References

[1] Wiersinga W.J., Rhodes A., Cheng A.C., Peacock S.J., Prescott H.C. (2020). Pathophysiology, Transmission, Diagnosis, and Treatment of Coronavirus Disease 2019 (COVID-19): A Review. JAMA.

[2] Zheng Z., Peng F., Xu B., Zhao J., Liu H., Peng J., Li Q., Jiang C., Zhou Y., Liu S. (2020). Risk factors of critical & mortal COVID-19 cases: A systematic literature review and meta-analysis. J. Infect..

[3] Boban M. (2021). Novel coronavirus disease (COVID-19) update on epidemiology, pathogenicity, clinical course and treatments. Int. J. Clin. Pract..

[4] Gavriatopoulou M., Ntanasis-Stathopoulos I., Korompoki E., Fotiou D., Migkou M., Tzanninis I.-G., Psaltopoulou T., Kastritis E., Terpos E., Dimopoulos M.A. (2021). Emerging treatment strategies for COVID-19 infection. Clin. Exp. Med..

[5] Brian D.A., Baric R.S. (2005). Coronavirus Genome Structure and Replication. Curr. Top Microbiol. Immunol..

[6] Li F. (2016). Structure, Function, and Evolution of Coronavirus Spike Proteins. Annu. Rev. Virol..

[7] Walls A.C., Park Y.J., Tortorici M.A., Wall A., McGuire A.T., Veesler D. (2020). Structure, Function, and Antigenicity of the SARS-CoV-2 Spike Glycoprotein. Cell.

[8] Yang G. (2020). H(2)S as a potential defense against COVID-19?. Am. J. Physiol. Cell Physiol..

[9] Huang Y., Yang C., Xu X.F., Xu W., Liu S.W. (2020). Structural and functional properties of SARS-CoV-2 spike protein: Potential antivirus drug development for COVID-19. Acta Pharmacol. Sin..

[10] Shen L.W., Mao H.J., Wu Y.L., Tanaka Y., Zhang W. (2017). TMPRSS2: A potential target for treatment of influenza virus and coronavirus infections. Biochimie.

[11] Hoffmann M., Kleine-Weber H., Schroeder S., Krüger N., Herrler T., Erichsen S., Schiergens T.S., Herrler G., Wu N.H., Nitsche A. (2020). SARS-CoV-2 Cell Entry Depends on ACE2 and TMPRSS2 and Is Blocked by a Clinically Proven Protease Inhibitor. Cell.

[12] Lukassen S., Chua R.L., Trefzer T., Kahn N.C., Schneider M.A., Muley T., Winter H., Meister M., Veith C., Boots A.W. (2020). SARS -CoV-2 receptor ACE 2 and TMPRSS 2 are primarily expressed in bronchial transient secretory cells. EMBO J..

[13] Imai Y., Kuba K., Rao S., Huan Y., Guo F., Guan B., Yang P., Sarao R., Wada T., Leong-Poi H. (2005). Angiotensin-converting enzyme 2 protects from severe acute lung failure. Nature.

[14] Danser A.H.J., Epstein M., Batlle D. (2020). Renin-Angiotensin System Blockers and the COVID-19 Pandemic: At Present There Is No Evidence to Abandon Renin-Angiotensin System Blockers. Hypertension.

[15] Amin S.A., Banerjee S., Ghosh K., Gayen S., Jha T. (2021). Protease targeted COVID-19 drug discovery and its challenges: Insight into viral main protease (Mpro) and papain-like protease (PLpro) inhibitors. Bioorgan. Med. Chem..

[16] Wang M.-Y., Zhao R., Gao L.-J., Gao X.-F., Wang D.-P., Cao J.-M. (2020). SARS-CoV-2: Structure, Biology, and Structure-Based Therapeutics Development. Front. Cell. Infect. Microbiol..

[17] Yang H., Xie W., Xue X., Yang K., Ma J., Liang W., Zhao Q., Zhou Z., Pei D., Ziebuhr J. (2005). Design of wide-spectrum inhibitors targeting coronavirus main proteases. PLoS Biol..

[18] Chan A.H., Schroder K. (2020). Inflammasome signaling and regulation of interleukin-1 family cytokines. J. Exp. Med..

[19] Ma C., Hu Y., Townsend J.A., Lagarias P.I., Marty M.T., Kolocouris A., Wang J. (2020). Ebselen, Disulfiram, Carmofur, PX-12, Tideglusib, and Shikonin Are Nonspecific Promiscuous SARS-CoV-2 Main Protease Inhibitors. ACS Pharmacol. Transl. Sci..

[20] Ferreira F.L., Bota D.P., Bross A., Melot C., Vincent J.-L. (2001). Serial Evaluation of the SOFA Score to Predict Outcome in Critically Ill Patients. JAMA.

[21] Shibuya N., Koike S., Tanaka M., Ishigami-Yuasa M., Kimura Y., Ogasawara Y., Fukui K., Nagahara N., Kimura H. (2013). A novel pathway for the production of hydrogen sulfide from D-cysteine in mammalian cells. Nat. Commun..

[22] Tomasova L., Konopelski P., Ufnal M. (2016). Gut Bacteria and Hydrogen Sulfide: The New Old Players in Circulatory System Homeostasis. Molecules.

[23] Rey F.E., Gonzalez M.D., Cheng J., Wu M., Ahern P.P., Gordon J.I. (2013). Metabolic niche of a prominent sulfate-reducing human gut bacterium. Proc. Natl. Acad. Sci. USA.

[24] Bouillaud F., Blachier F. (2011). Mitochondria and Sulfide: A Very Old Story of Poisoning, Feeding, and Signaling?. Antioxid. Redox Signal..

[25] Jiang J., Chan A., Ali S.S., Saha A., Haushalter K.J., Lam W.-L.M., Glasheen M., Parker J., Brenner M., Mahon S.B. (2016). Hydrogen Sulfide—Mechanisms of Toxicity and Development of an Antidote. Sci. Rep..

[26] Hildebrandt T.M., Grieshaber M.K. (2008). Three enzymatic activities catalyze the oxidation of sulfide to thiosulfate in mammalian and invertebrate mitochondria. FEBS J..

[27] Toohey J.I. (2011). Sulfur signaling: Is the agent sulfide or sulfane?. Anal. Biochem..

[28] Iciek M., Kowalczyk-Pachel D., Bilska-Wilkosz A., Kwiecień I., Górny M., Włodek L. (2015). S-sulfhydration as a cellular redox regulation. Biosci. Rep..

[29] Kumar P., Osahon O., Vides D.B., Hanania N., Minard C.G., Sekhar R.V. (2021). Severe Glutathione Deficiency, Oxidative Stress and Oxidant Damage in Adults Hospitalized with COVID-19: Implications for GlyNAC (Glycine and N-Acetylcysteine) Supplementation. Antioxidants.

[30] Yang G., Wu L., Jiang B., Yang W., Qi J., Cao K., Meng Q., Mustafa A.K., Mu W., Zhang S. (2008). H2S as a physiologic vasorelaxant: Hypertension in mice with deletion of cystathionine gamma-lyase. Science.

[31] Lobb I., Sonke E., Aboalsamh G., Sener A. (2015). Hydrogen sulphide and the kidney: Important roles in renal physiology and pathogenesis and treatment of kidney injury and disease. Nitric Oxide.

[32] Dugbartey G.J. (2018). The smell of renal protection against chronic kidney disease: Hydrogen sulfide offers a potential stinky remedy. Pharmacol. Rep..

[33] Szabo C. (2012). Roles of Hydrogen Sulfide in the Pathogenesis of Diabetes Mellitus and Its Complications. Antioxid. Redox. Signal..

[34] Sun H.-J., Wu Z.-Y., Cao L., Zhu M.-Y., Liu T.-T., Guo L., Lin Y., Nie X.-W., Bian J.-S. (2019). Hydrogen Sulfide: Recent Progression and Perspectives for the Treatment of Diabetic Nephropathy. Molecules.

[35] Wallace J.L., Wang R. (2015). Hydrogen sulfide-based therapeutics: Exploiting a unique but ubiquitous gasotransmitter. Nat. Rev. Drug Discov..

[36] Lin Y., Zeng H., Gao L., Gu T., Wang C., Zhang H. (2017). Hydrogen Sulfide Attenuates Atherosclerosis in a Partially Ligated Carotid Artery Mouse model via Regulating Angiotensin Converting Enzyme 2 Expression. Front. Physiol..

[37] Pozzi G., Masselli E., Gobbi G., Mirandola P., Taborda-Barata L., Ampollini L., Carbognani P., Micheloni C., Corazza F., Galli D. (2021). Hydrogen Sulfide Inhibits TMPRSS2 in Human Airway Epithelial Cells: Implications for SARS-CoV-2 Infection. Biomedicines.

[38] Zhao K., Li S., Wu L., Lai C., Yang G. (2014). Hydrogen Sulfide Represses Androgen Receptor Transactivation by Targeting at the Second Zinc Finger Module. J. Biol. Chem..

[39] Bazhanov N., Escaffre O., Freiberg A.N., Garofalo R.P., Casola A. (2017). Broad-Range Antiviral Activity of Hydrogen Sulfide Against Highly Pathogenic RNA Viruses. Sci. Rep..

[40] Pal V.K., Bandyopadhyay P., Singh A. (2018). Hydrogen sulfide in physiology and pathogenesis of bacteria and viruses. IUBMB Life.

[41] Li H., Ma Y., Escaffre O., Ivanciuc T., Komaravelli N., Kelley J.P., Coletta C., Szabo C., Rockx B., Garofalo R.P. (2015). Role of Hydrogen Sulfide in Paramyxovirus Infections. J. Virol..

[42] Bazhanov N., Ivanciuc T., Wu H., Garofalo M., Kang J., Xian M., Casola A. (2018). Thiol-Activated Hydrogen Sulfide Donors Antiviral and Anti-Inflammatory Activity in Respiratory Syncytial Virus Infection. Viruses.

[43] Ivanciuc T., Sbrana E., Casola A., Garofalo R.P. (2019). Cystathionine γ-lyase deficiency enhances airway reactivity and viral-induced disease in mice exposed to side-stream tobacco smoke. Pediatr. Res..

[44] Coperchini F., Chiovato L., Croce L., Magri F., Rotondi M. (2020). The cytokine storm in COVID-19: An overview of the involvement of the chemokine/chemokine-receptor system. Cytokine Growth Factor Rev..

[45] Citi V., Martelli A., Brancaleone V., Brogi S., Gojon G., Montanaro R., Morales G., Testai L., Calderone V. (2020). Anti-inflammatory and antiviral roles of hydrogen sulfide: Rationale for considering H_2_S donors in COVID-19 therapy. Br. J. Pharm..

[46] Conti P., Ronconi G., Caraffa A., Gallenga C.E., Ross R., Frydas I., Kritas S.K. (2020). Induction of pro-inflammatory cytokines (IL-1 and IL-6) and lung inflammation by Coronavirus-19 (COVI-19 or SARS-CoV-2): Anti-inflammatory strategies. J. Biol. Regul. Homeost Agents.

[47] Gubernatorova E.O., Gorshkova E.A., Polinova A.I., Drutskaya M.S. (2020). IL-6: Relevance for immunopathology of SARS-CoV-2. Cytokine Growth Factor. Rev..

[48] Li T., Zhao B., Wang C., Wang H., Liu Z., Li W., Jin H., Tang C., Du J. (2008). Regulatory Effects of Hydrogen Sulfide on IL-6, IL-8 and IL-10 Levels in the Plasma and Pulmonary Tissue of Rats with Acute Lung Injury. Exp. Biol. Med..

[49] Kloesch B., Liszt M., Broell J. (2010). H2S transiently blocks IL-6 expression in rheumatoid arthritic fibroblast-like synoviocytes and deactivates p44/42 mitogen-activated protein kinase. Cell Biol. Int..

[50] Faller S., Hausler F., Goeft A., Von Itter M.-N.A., Gyllenram V., Hoetzel A., Spassov S.G. (2018). Hydrogen sulfide limits neutrophil transmigration, inflammation, and oxidative burst in lipopolysaccharide-induced acute lung injury. Sci. Rep..

[51] Shin I.S., Hong J., Jeon C.M., Shin N.R., Kwon O.K., Kim H.S., Kim J.C., Oh S.R., Ahn K.S. (2013). Diallyl-disulfide, an organosulfur compound of garlic, attenuates airway inflammation via activation of the Nrf-2/HO-1 pathway and NF-kappaB suppression. Food Chem. Toxicol..

[52] Gasparello J., D’Aversa E., Papi C., Gambari L., Grigolo B., Borgatti M., Finotti A., Gambari R. (2021). Sulforaphane inhibits the expression of interleukin-6 and interleukin-8 induced in bronchial epithelial IB3-1 cells by exposure to the SARS-CoV-2 Spike protein. Phytomedicine.

[53] Renieris G., Katrini K., Damoulari C., Akinosoglou K., Psarrakis C., Kyriakopoulou M., Dimopoulos G., Lada M., Koufargyris P., Giamarellos-Bourboulis E.J. (2020). Serum Hydrogen Sulfide and Outcome Association in Pneumonia by the SARS-CoV-2 Coronavirus. Shock.

[54] Zhang D., Wang X., Chen S., Chen S., Yu W., Liu X., Yang G., Tao Y., Tang X., Bu D. (2019). Endogenous hydrogen sulfide sulfhydrates IKKβ at cysteine 179 to control pulmonary artery endothelial cell inflammation. Clin. Sci..

[55] Dosch S.F., Mahajan S.D., Collins A.R. (2009). SARS coronavirus spike protein-induced innate immune response occurs via activation of the NF-kappaB pathway in human monocyte macrophages in vitro. Virus Res..

[56] Catanzaro M., Fagiani F., Racchi M., Corsini E., Govoni S., Lanni C. (2020). Immune response in COVID-19: Addressing a pharmacological challenge by targeting pathways triggered by SARS-CoV-2. Signal Transduct. Target. Ther..

[57] Castelblanco M., Lugrin J., Ehirchiou D., Nasi S., Ishii I., So A., Martinon F., Busso N. (2018). Hydrogen sulfide inhibits NLRP3 inflammasome activation and reduces cytokine production both in vitro and in a mouse model of inflammation. J. Biol. Chem..

[58] Yue L.-M., Gao Y.-M., Han B.-H. (2019). Evaluation on the effect of hydrogen sulfide on the NLRP3 signaling pathway and its involvement in the pathogenesis of atherosclerosis. J. Cell. Biochem..

[59] Soto M.E., Guarner-Lans V., Díaz-Díaz E., Manzano-Pech L., Palacios-Chavarría A., Valdez-Vázquez R.R., Aisa-Álvarez A., Saucedo-Orozco H., Pérez-Torres I. (2022). Hyperglycemia and Loss of Redox Homeostasis in COVID-19 Patients. Cells.

[60] Janssen W.J., Stefanski A.L., Bochner B.S., Evans C.M. (2016). Control of lung defence by mucins and macrophages: Ancient defence mechanisms with modern functions. Eur. Respir. J..

[61] Viegas J., Esteves A.F., Cardoso E.M., Arosa F.A., Vitale M., Taborda-Barata L. (2019). Biological Effects of Thermal Water-Associated Hydrogen Sulfide on Human Airways and Associated Immune Cells: Implications for Respiratory Diseases. Front. Public Health.

[62] Pouokam E., Althaus M. (2016). Epithelial Electrolyte Transport Physiology and the Gasotransmitter Hydrogen Sulfide. Oxidative Med. Cell. Longev..

[63] Dominic P., Ahmad J., Bhandari R., Pardue S., Solorzano J., Jaisingh K., Watts M., Bailey S.R., Orr A.W., Kevil C.G. (2021). Decreased availability of nitric oxide and hydrogen sulfide is a hallmark of COVID-19. Redox Biol..

[64] Boman G., Bäcker U., Larsson S., Melander B., Wåhlander L. (1983). Oral acetylcysteine reduces exacerbation rate in chronic bronchitis: Report of a trial organized by the Swedish Society for Pulmonary Diseases. Eur. J. Respir. Dis..

[65] Benlamkaddem S., Iken I., Houari N., Elbouazzaoui A., Boukatta B., Sbai H., Achour S., Kanjaa N. (2018). Paracetamol self-poisoning: When oral N-acetylcysteine saves life? A case report. Pan. Afr. Med. J..

[66] Berk M., Malhi G.S., Gray L.J., Dean O.M. (2013). The promise of N-acetylcysteine in neuropsychiatry. Trends Pharmacol. Sci..

[67] Minarini A., Ferrari S., Galletti M., Giambalvo N., Perrone D., Rioli G., Galeazzi G.M. (2017). *N*-acetylcysteine in the treatment of psychiatric disorders: Current status and future prospects. Expert Opin. Drug Metab. Toxicol..

[68] Slattery J., Kumar N., Delhey L., Berk M., Dean O., Spielholz C., Frye R. (2015). Clinical trials of N-acetylcysteine in psychiatry and neurology: A systematic review. Neurosci. Biobehav. Rev..

[69] Smaga I., Frankowska M., Filip M. (2021). N-acetylcysteine as a new prominent approach for treating psychiatric disorders. Br. J. Pharmacol..

[70] Smaga I., Frankowska M., Filip M. (2021). N-acetylcysteine in substance use disorder: A lesson from preclinical and clinical research. Pharmacol. Rep..

[71] Skvarc D.R., Dean O.M., Byrne L.K., Gray L.J., Ives K., Lane S.E., Lewis M., Osborne C., Page R., Stupart D. (2016). The Post-Anaesthesia N-acetylcysteine Cognitive Evaluation (PANACEA) trial: Study protocol for a randomised controlled trial. Trials.

[72] Skvarc D., Dean O.M., Byrne L.K., Gray L., Lane S., Lewis M., Fernandes B., Berk M., Marriott A. (2017). The effect of N-acetylcysteine (NAC) on human cognition–A systematic review. Neurosci. Biobehav. Rev..

[73] Siu J.T., Tejani A.M., Nguyen T., Turgeon R.D. (2020). N-acetylcysteine for non-paracetamol (acetaminophen)-related acute liver failure. Cochrane Database Syst. Rev..

[74] Ghafarizadeh A., Malmir M., Noreini S.N., Faraji T. (2021). Antioxidant effects of N-acetylcysteine on the male reproductive system: A systematic review. Andrologia.

[75] Adil M., Amin S.S., Mohtashim M. (2018). N-acetylcysteine in dermatology. Indian J. Derm. Venereol. Leprol..

[76] Geiler J., Michaelis M., Naczk P., Leutz A., Langer K., Doerr H.-W., Cinatl J. (2010). N-acetyl-l-cysteine (NAC) inhibits virus replication and expression of pro-inflammatory molecules in A549 cells infected with highly pathogenic H5N1 influenza A virus. Biochem. Pharmacol..

[77] Arranz L., Fernández C., Rodríguez A., Ribera J.M., De la Fuente M. (2008). The glutathione precursor N-acetylcysteine improves immune function in postmenopausal women. Free Radic. Biol. Med..

[78] Savva D.A., Crist M., Lardieri A. (2019). N-Acetylcysteine for Gastric Lactobezoars in a 1-Month-Old. J. Pediatr. Pharmacol. Ther..

[79] Ibrahim H., Perl A., Smith D., Lewis T., Kon Z., Goldenberg R., Yarta K., Staniloae C., Williams M. (2020). Therapeutic blockade of inflammation in severe COVID-19 infection with intravenous N-acetylcysteine. Clin. Immunol..

[80] Assimakopoulos S.F., Aretha D., Komninos D., Dimitropoulou D., Lagadinou M., Leonidou L., Oikonomou I., Mouzaki A., Marangos M. (2021). N-acetyl-cysteine reduces the risk for mechanical ventilation and mortality in patients with COVID-19 pneumonia: A two-center retrospective cohort study. Infect. Dis..

[81] De Flora S., Balansky R., La Maestra S. (2020). Rationale for the use of N-acetylcysteine in both prevention and adjuvant therapy of COVID-19. FASEB J..

[82] Shi Z., Puyo C.A. (2020). N-Acetylcysteine to Combat COVID-19: An Evidence Review. Ther. Clin. Risk Manag..

[83] Abdolrazaghnejad A., Sharafkhah M., Zarinfar N., Mohammadbeigi A., Massoudifar A., Abaszadeh S. (2018). Safety and efficacy of N-acetyl-cysteine for prophylaxis of ventilator-associated pneumonia: A randomized, double blind, placebo-controlled clinical trial. Med. Gas Res..

[84] Suter P.M., Domenighetti G., Schaller M.D., Laverrière M.C., Ritz R., Perret C. (1994). N-acetylcysteine enhances recovery from acute lung injury in man. A randomized, double-blind, placebo-controlled clinical study. Chest.

[85] Lei Q., Yi T., Chen C. (2018). NF-κB-Gasdermin D (GSDMD) Axis Couples Oxidative Stress and NACHT, LRR and PYD Domains-Containing Protein 3 (NLRP3) Inflammasome-Mediated Cardiomyocyte Pyroptosis Following Myocardial Infarction. Med. Sci. Monit..

[86] Ehre C., Rushton Z.L., Wang B., Hothem L.N., Morrison C.B., Fontana N.C., Markovetz M.R., Delion M.F., Kato T., Villalon D. (2019). An Improved Inhaled Mucolytic to Treat Airway Muco-obstructive Diseases. Am. J. Respir. Crit. Care Med..

[87] Pedre B., Barayeu U., Ezeriņa D., Dick T.P. (2021). The mechanism of action of N-acetylcysteine (NAC): The emerging role of H(2)S and sulfane sulfur species. Pharmacol. Ther..

[88] Gleixner A.M., Hutchison D.F., Sannino S., Bhatia T.N., Leak L.C., Flaherty P.T., Wipf P., Brodsky J.L., Leak R.K. (2017). *N*-Acetyl-l-Cysteine Protects Astrocytes against Proteotoxicity without Recourse to Glutathione. Mol. Pharmacol..

[89] Ezeriņa D., Takano Y., Hanaoka K., Urano Y., Dick T.P. (2018). N-Acetyl Cysteine Functions as a Fast-Acting Antioxidant by Triggering Intracellular H. Cell Chem. Biol..

[90] Bourgonje A.R., Offringa A.K., van Eijk L.E., Abdulle A.E., Hillebrands J.L., van der Voort P.H., van Goor H., van Hezik E.J. (2021). N-Acetylcysteine and Hydrogen Sulfide in Coronavirus Disease 2019. Antioxid. Redox. Signal.

[91] Fukuto J.M., Ignarro L.J., Nagy P., Wink D.A., Kevil C.G., Feelisch M., Cortese-Krott M.M., Bianco C.L., Kumagai Y., Hobbs A.J. (2018). Biological hydropersulfides and related polysulfides—A new concept and perspective in redox biology. FEBS Lett..

[92] Wang Y., Dillon K.M., Li Z., Winckler E.W., Matson J.B. (2020). Alleviating Cellular Oxidative Stress through Treatment with Superoxide-Triggered Persulfide Prodrugs. Angew. Chem. Int. Ed. Engl..

[93] Yuan Z., Zheng Y., Yu B., Wang S., Yang X., Wang B. (2018). Esterase-Sensitive Glutathione Persulfide Donor. Org. Lett..

[94] Gonet-Surówka A., Dynarowicz-Łątka P. (2021). From glioblastoma to COVID-19–role of oxysterols in the human organism. Postepy Biochem..

[95] Marcello A., Civra A., Bonotto R.M., Alves L.N., Rajasekharan S., Giacobone C., Caccia C., Cavalli R., Adami M., Brambilla P. (2020). The cholesterol metabolite 27-hydroxycholesterol inhibits SARS-CoV-2 and is markedly decreased in COVID-19 patients. Redox Biol..

[96] Ghzaiel I., Sassi K., Zarrouk A., Nury T., Ksila M., Leoni V., Bouhaouala-Zahar B., Hammami S., Hammami M., Mackrill J.J. (2021). 7-Ketocholesterol: Effects on viral infections and hypothetical contribution in COVID-19. J. Steroid Biochem. Mol. Biol..

[97] Pawlos A., Niedzielski M., Gorzelak-Pabiś P., Broncel M., Woźniak E. (2021). COVID-19, Direct and Indirect Mechanisms of Statins. Int. J. Mol. Sci..

[98] Nury T., Yammine A., Ghzaiel I., Sassi K., Zarrouk A., Brahmi F., Samadi M., Rup-Jacques S., Vervandier-Fasseur D., de Barros J.P. (2021). Attenuation of 7-ketocholesterol- and 7β-hydroxycholesterol-induced oxiapoptophagy by nutrients, synthetic molecules and oils: Potential for the prevention of age-related diseases. Ageing Res. Rev..

[99] Trevisi L., Bertoldo A., Agnoletto L., Poggiani C., Cusinato F., Luciani S. (2010). Antiapoptotic and Proliferative Effects of Low Concentrations of 7β-Hydroxycholesterol in Human Endothelial Cells via ERK Activation. J. Vasc. Res..

[100] Wang S.-F., Chou Y.-C., Mazumder N., Kao F.-J., Nagy L.D., Guengerich F.P., Huang C., Lee H.-C., Lai P.-S., Ueng Y.-F. (2013). 7-Ketocholesterol induces P-glycoprotein through PI3K/mTOR signaling in hepatoma cells. Biochem. Pharmacol..

[101] Lizard G., Gueldry S., Sordet O., Monier S., Athias A., Miguet C., Bessede G., Lemaire S., Solary E., Gambert P. (1998). Glutathione is implied in the control of 7-ketocholesterol-induced apoptosis, which is associated with radical oxygen species production. Faseb J..

[102] Anderson A., Campo A., Fulton E., Corwin A., Jerome W.G., O’Connor M.S. (2020). 7-Ketocholesterol in disease and aging. Redox Biol..

[103] Vasiliou V., Nebert D.W. (2005). Analysis and update of the human aldehyde dehydrogenase (ALDH) gene family. Hum. Genom..

[104] Ekinci E., Rohondia S., Khan R., Dou Q.P. (2019). Repurposing Disulfiram as An Anti-Cancer Agent: Updated Review on Literature and Patents. Recent Pat. Anti-Cancer Drug Discov..

[105] Shirley D.-A., Sharma I., Warren C.A., Moonah S. (2021). Drug Repurposing of the Alcohol Abuse Medication Disulfiram as an Anti-Parasitic Agent. Front. Cell. Infect. Microbiol..

[106] Elliott J.H., McMahon J., Chang C.C., Lee S.A., Hartogensis W., Bumpus N., Savic R., Roney J., Hoh R., Solomon A. (2015). Short-term administration of disulfiram for reversal of latent HIV infection: A phase 2 dose-escalation study. Lancet HIV.

[107] Lin M.-H., Moses D.C., Hsieh C.-H., Cheng S.-C., Chen Y.-H., Sun C.-Y., Chou C.-Y. (2017). Disulfiram can inhibit MERS and SARS coronavirus papain-like proteases via different modes. Antivir. Res..

[108] Tamburin S., Mantovani E., De Bernardis E., Zipeto D., Lugoboni F., Agostoni C., Almasio R., Avveduti P., Baisini O., Ballerio M. (2021). COVID-19 and related symptoms in patients under disulfiram for alcohol use disorder. Intern. Emerg. Med..

[109] Fillmore N., Bell S., Shen C., Nguyen V., La J., Dubreuil M., Strymish J., Brophy M., Mehta G., Wu H. (2021). Disulfiram use is associated with lower risk of COVID-19: A retrospective cohort study. PLoS ONE.

[110] Jin Z., Du X., Xu Y., Deng Y., Liu M., Zhao Y., Zhang B., Li X., Zhang L., Peng C. (2020). Structure of M(pro) from SARS-CoV-2 and discovery of its inhibitors. Nature.

[111] Amatore D., Celestino I., Brundu S., Galluzzi L., Coluccio P., Checconi P., Magnani M., Palamara A.T., Fraternale A., Nencioni L. (2019). Glutathione increase by the n-butanoyl glutathione derivative (GSH-C4) inhibits viral replication and induces a predominant Th1 immune profile in old mice infected with influenza virus. FASEB BioAdvances.

[112] Lobo-Galo N., Terrazas-López M., Martínez-Martínez A., Díaz-Sánchez Á. (2021). FDA-approved thiol-reacting drugs that potentially bind into the SARS-CoV-2 main protease, essential for viral replication. J. Biomol. Struct. Dyn..

[113] Hu J.J., Liu X., Xia S., Zhang Z., Zhang Y., Zhao J., Ruan J., Luo X., Lou X., Bai Y. (2020). FDA-approved disulfiram inhibits pyroptosis by blocking gasdermin D pore formation. Nat. Immunol..

[114] Adrover J.M., Carrau L., Daßler-Plenker J., Bram Y., Chandar V., Houghton S., Redmond D., Merrill J.R., Shevik M. (2022). Disulfiram inhibits neutrophil extracellular trap formation and protects rodents from acute lung injury and SARS-CoV-2 infection. JCI Insight.

[115] Buckingham S.C., Jafri H.S., Bush A.J., Carubelli C.M., Sheeran P., Hardy R.D., Ottolini M.G., Ramilo O., DeVincenzo J.P. (2002). A randomized, double-blind, placebo-controlled trial of dexamethasone in severe respiratory syncytial virus (RSV) infection: Effects on RSV quantity and clinical outcome. J. Infect. Dis..

[116] Biewenga G.P., Haenen G.R.M.M., Bast A. (1997). The pharmacology of the antioxidant lipoic acid. Gen. Pharmacol..

[117] Bilska A., Włodek L. (2005). Lipoic acid-the drug of the future?. Pharmacol. Rep..

[118] Packer L., Witt E.H., Tritschler H.J. (1995). Alpha-lipoic acid as a biological antioxidant. Free Radic. Biol. Med..

[119] Goraca A., Skibska B. (2008). Beneficial effect of alpha-lipoic acid on lipopolysaccharide-induced oxidative stress in bronchoalveolar lavage fluid. J. Physiol. Pharmacol..

[120] Liu Z., Guo J., Sun H., Huang Y., Zhao R., Yang X. (2015). α-Lipoic acid attenuates LPS-induced liver injury by improving mitochondrial function in association with GR mitochondrial DNA occupancy. Biochimie.

[121] Kwiecień B., Dudek M., Bilska-Wilkosz A., Knutelska J., Bednarski M., Kwiecień I., Zygmunt M., Iciek M., Sokołowska-Jeżewicz M., Sapa J. (2013). In vivo anti-inflammatory activity of lipoic acid derivatives in mice. Postepy Hig. Med. Dosw. (Online).

[122] Jariwalla R.J., Lalezari J., Cenko D., Mansour S.E., Kumar A., Gangapurkar B., Nakamura D. (2008). Restoration of blood total glutathione status and lymphocyte function following alpha-lipoic acid supplementation in patients with HIV infection. J. Altern. Complement Med..

[123] Berkson B.M. (1999). A conservative triple antioxidant approach to the treatment of hepatitis C. Combination of alpha lipoic acid (thioctic acid), silymarin, and selenium: Three case histories. Med. Klin..

[124] Shi C., Sun Y., Zhang X., Zheng Z., Yang M., Ben H., Song K., Cao Y., Chen Y., Liu X. (2016). Antimicrobial effect of lipoic acid against Cronobacter sakazakii. Food Control..

[125] Biernat-Sudolska M., Rojek-Zakrzewska D., Bilska-Wilkosz A. (2020). In-vitro activity of lipoic acid against Ureaplasma urealyticum and Ureaplasma parvum isolated from women with infections of the urogenital tract. A pilot study. Acta Biochim. Pol..

[126] Petersen Shay K., Moreau R.F., Smith E.J., Hagen T.M. (2008). Is alpha-lipoic acid a scavenger of reactive oxygen species in vivo? Evidence for its initiation of stress signaling pathways that promote endogenous antioxidant capacity. IUBMB Life.

[127] Rochette L., Ghibu S. (2021). Mechanics Insights of Alpha-Lipoic Acid against Cardiovascular Diseases during COVID-19 Infection. Int. J. Mol. Sci..

[128] Dragomanova S., Miteva S., Nicoletti F., Mangano K., Fagone P., Pricoco S., Staykov H., Tancheva L. (2021). Therapeutic Potential of Alpha-Lipoic Acid in Viral Infections, including COVID-19. Antioxidants.

[129] Jalilpiran Y., Hajishafiee M., Khorshidi M., Rezvani H., Mohammadi-Sartang M., Rahmani J., Mousavi S.M. (2020). The effect of Alpha-lipoic acid supplementation on endothelial function: A systematic review and meta-analysis. Phytother. Res..

[130] Petri S., Körner S., Kiaei M. (2012). Nrf2/ARE Signaling Pathway: Key Mediator in Oxidative Stress and Potential Therapeutic Target in ALS. Neurol. Res. Int..

[131] Zhang J., Zhou X., Wu W., Wang J., Xie H., Wu Z. (2017). Regeneration of glutathione by α-lipoic acid via Nrf2/ARE signaling pathway alleviates cadmium-induced HepG2 cell toxicity. Env. Toxicol Pharm..

[132] Zhong M., Sun A., Xiao T., Yao G., Sang L., Zheng X., Zhang J., Jin X., Xu L., Yang W. (2021). A Randomized, Single-Blind, Group Sequential, Active-Controlled Study to Evaluate the Clinical Efficacy and Safety of α-Lipoic Acid for Critically Ill Patients With Coronavirus Disease 2019 (COVID-19). Front. Med..

[133] Hummel T., Heilmann S., Hüttenbriuk K.-B. (2002). Lipoic Acid in the Treatment of Smell Dysfunction Following Viral Infection of the Upper Respiratory Tract. Laryngoscope.

[134] Sayıner S., Şehirli A., Serakıncı N. (2020). Alpha Lipoic Acid as a Potential Treatment for COVID-19-A Hypothesis. Curr. Top. Nutraceutical Res..

[135] Uberti F., Ruga S., Farghali M., Galla R., Molinari C. (2021). A Combination of α-Lipoic Acid (ALA) and Palmitoylethanolamide (PEA) Blocks Endotoxin-Induced Oxidative Stress and Cytokine Storm: A Possible Intervention for COVID-19. J. Diet. Suppl..

[136] Dudek M., Razny K., Bilska-Wilkosz A., Iciek M., Sapa J., Wlodek L., Filipek B. (2016). Hypotensive effect of alpha-lipoic acid after a single administration in rats. Anatol. J. Cardiol..

[137] Dudek M., Knutelska J., Bednarski M., Nowiński L., Zygmunt M., Bilska-Wilkosz A., Iciek M., Otto M., Żytka I., Sapa J. (2014). Alpha lipoic acid protects the heart against myocardial post ischemia–reperfusion arrhythmias via KATP channel activation in isolated rat hearts. Pharmacol. Rep..

[138] Cure E., Cure M.C. (2020). Alpha-lipoic acid may protect patients with diabetes against COVID-19 infection. Med. Hypotheses.

[139] Mccarty M.F., Iloki Assanga S.B., Luján L.L., O’Keefe J.H., DiNicolantonio J.J. (2020). Nutraceutical Strategies for Suppressing NLRP3 Inflammasome Activation: Pertinence to the Management of COVID-19 and Beyond. Nutrients.

[140] Mikami Y., Shibuya N., Kimura Y., Nagahara N., Ogasawara Y., Kimura H. (2011). Thioredoxin and dihydrolipoic acid are required for 3-mercaptopyruvate sulfurtransferase to produce hydrogen sulfide. Biochem. J..

[141] Bilska A., Dudek M., Iciek M., Kwiecień I., Sokołowska-Jezewicz M., Filipek B., Włodek L. (2008). Biological actions of lipoic acid associated with sulfane sulfur metabolism. Pharm. Rep..

[142] Dudek M., Bilska-Wilkosz A., Knutelska J., Mogilski S., Bednarski M., Zygmunt M., Iciek M., Sapa J., Bugajski D., Filipek B. (2013). Are anti-inflammatory properties of lipoic acid associated with the formation of hydrogen sulfide?. Pharm. Rep..

[143] Bilska-Wilkosz A., Iciek M., Kowalczyk-Pachel D., Górny M., Sokołowska-Jeżewicz M., Włodek L. (2017). Lipoic Acid as a Possible Pharmacological Source of Hydrogen Sulfide/Sulfane Sulfur. Molecules.

[144] Forman H.J., Zhang H., Rinna A. (2009). Glutathione: Overview of its protective roles, measurement, and biosynthesis. Mol. Asp. Med..

[145] Dröge W. (2002). Aging-related changes in the thiol/disulfide redox state: Implications for the use of thiol antioxidants. Exp. Gerontol..

[146] Robaczewska J., Kedziora-Kornatowska K., Kozakiewicz M., Zary-Sikorska E., Pawluk H., Pawliszak W., Kedziora J. (2016). Role of glutathione metabolism and glutathione-related antioxidant defense systems in hypertension. J. Physiol. Pharmacol..

[147] Musthafa Q.A., Shukor M.F.A., Ismail N.A.S., Ghazi A.M., Ali R.M., Nor I.F.M., Dimon M.Z., Ngah W.Z.W. (2017). Oxidative status and reduced glutathione levels in premature coronary artery disease and coronary artery disease. Free Radic. Res..

[148] Lutchmansingh F.K., Hsu J.W., Bennett F.I., Badaloo A., McFarlane-Anderson N., Gordon-Strachan G.M., Wright-Pascoe R.A., Jahoor F., Boyne M.S. (2018). Glutathione metabolism in type 2 diabetes and its relationship with microvascular complications and glycemia. PLoS ONE.

[149] Khan N.I., Naz L., Yasmeen G. (2006). Obesity: An independent risk factor for systemic oxidative stress. Pak. J. Pharm. Sci..

[150] Moriarty S.E., Shah J.H., Lynn M., Jiang S., Openo K., Jones D.P., Sternberg P. (2003). Oxidation of glutathione and cysteine in human plasma associated with smoking. Free Radic. Biol. Med..

[151] Khanfar A., Al Qaroot B. (2020). Could glutathione depletion be the Trojan horse of COVID-19 mortality?. Eur. Rev. Med. Pharmacol. Sci..

[152] Wang J., Chen Y., Gao N., Wang Y., Tian Y., Wu J., Zhang J., Zhu J., Fan D., An J. (2013). Inhibitory Effect of Glutathione on Oxidative Liver Injury Induced by Dengue Virus Serotype 2 Infections in Mice. PLoS ONE.

[153] Palamara A.T., Perno C.F., Ciriolo M.R., Dini L., Balestra E., D’Agostini C., Di Francesco P., Favalli C., Rotilio G., Garaci E. (1995). Evidence for antiviral activity of glutathione: In vitro inhibition of herpes simplex virus type 1 replication. Antivir. Res..

[154] Khomich O.A., Kochetkov S.N., Bartosch B., Ivanov A.V. (2018). Redox Biology of Respiratory Viral Infections. Viruses.

[155] Bartolini D., Stabile A.M., Bastianelli S., Giustarini D., Pierucci S., Busti C., Vacca C., Gidari A., Francisci D., Castronari R. (2021). SARS-CoV2 infection impairs the metabolism and redox function of cellular glutathione. Redox. Biol..

[156] Sekhar R.V., Patel S.G., Guthikonda A.P., Reid M., Balasubramanyam A., Taffet G.E., Jahoor F. (2011). Deficient synthesis of glutathione underlies oxidative stress in aging and can be corrected by dietary cysteine and glycine supplementation. Am. J. Clin. Nutr..

[157] Nguyen D., Hsu J.W., Jahoor F., Sekhar R.V. (2014). Effect of increasing glutathione with cysteine and glycine supplementation on mitochondrial fuel oxidation, insulin sensitivity, and body composition in older HIV-infected patients. J. Clin. Endocrinol. Metab..

[158] Horowitz R., Phyllis F., James B. (2020). Efficacy of glutathione therapy in relieving dyspnea associated with COVID-19 pneumonia: A report of 2 cases. Respir. Med. Case Rep..

[159] Yammine A., Zarrouk A., Nury T., Vejux A., Latruffe N., Vervandier-Fasseur D., Samadi M., Mackrill J.J., Greige-Gerges H., Auezova L. (2020). Prevention by Dietary Polyphenols (Resveratrol, Quercetin, Apigenin) against 7-Ketocholesterol-Induced Oxiapoptophagy in Neuronal N2a Cells: Potential Interest for the Treatment of Neurodegenerative and Age-Related Diseases. Cells.

[160] Dechant K.L., Noble S. (1996). Erdosteine. Drugs.

[161] Cazzola M., Page C., Rogliani P., Calzetta L., Matera M.G. (2020). Multifaceted Beneficial Effects of Erdosteine: More than a Mucolytic Agent. Drugs.

[162] Dal S.M., Bovio C., Culici M., Braga P.C. (2002). The combination of the SH metabolite of erdosteine (a mucoactive drug) and ciprofloxacin increases the inhibition of bacterial adhesiveness achieved by ciprofloxacin alone. Drugs Exp. Clin. Res..

[163] Miyake K., Kaise T., Hosoe H., Akuta K., Manabe H., Ohmori K. (1999). The effect of erdosteine and its active metabolite on reactive oxygen species production by inflammatory cells. Arzneimittelforschung.

[164] Hosoe H., Kaise T., Ohmori K. (2002). Effects on the Reactive Oxygen Species of Erdosteine and its Metabolite in vitro. Arzneimittelforschung.

[165] Cazzola M., Calzetta L., Page C., Rogliani P., Matera M.G. (2018). Impact of erdosteine on chronic bronchitis and COPD: A meta-analysis. Pulm. Pharmacol. Ther..

[166] Recipharm’s Proprietary Molecule Erdosteine Has Been Positively Tested as Part of COVID-19 Treatment. https://mb.cision.com/Main/9273/3215696/1318818.pdf.

[167] Santus P., Tursi F., Croce G., Di Simone C., Frassanito F., Gaboardi P., Airoldi A., Pecis M., Negretto G., Radovanovic D. (2020). Changes in quality of life and dyspnoea after hospitalization in COVID-19 patients discharged at home. Multidiscip. Respir. Med..

[168] Dal Negro R.W., Visconti M., Micheletto C., Tognella S. (2008). Changes in blood ROS, e-NO, and some pro-inflammatory mediators in bronchial secretions following erdosteine or placebo: A controlled study in current smokers with mild COPD. Pulm. Pharm..

[169] Dal Negro R.W., Visconti M., Tognella S., Micheletto C. (2011). Erdosteine affects eicosanoid production in COPD. Int. J. Clin. Pharm..

[170] Braga P.C., Zuccotti T., Sasso M.D. (2001). Bacterial Adhesiveness: Effects of the SH Metabolite of Erdosteine (Mucoactive Drug) plus Clarithromycin versus Clarithromycin Alone. Chemotherapy.

[171] Pani A., Lucini V., Dugnani S., Scaglione F. (2022). Erdosteine enhances antibiotic activity against bacteria within biofilm. Int. J. Antimicrob. Agents.

[172] Kopriva F., Latalova V. (2019). Improving the Primary Care Management of Preschool Children with Recurrent Acute Respiratory Tract Infections in the Czech Republic: Prompt Use of Erdosteine Can Reduce Antibiotic Prescribing. Qual. Prim. Care.

[173] Tschirka J., Kreisor M., Betz J., Gründemann D. (2018). Substrate Selectivity Check of the Ergothioneine Transporter. Drug Metab. Dispos..

[174] Pochini L., Galluccio M., Scalise M., Console L., Pappacoda G., Indiveri C. (2022). OCTN1: A Widely Studied but Still Enigmatic Organic Cation Transporter Linked to Human Pathology and Drug Interactions. Int. J. Mol. Sci..

[175] Cheah I.K., Tang R.M.Y., Yew T.S.Z.Z., Lim K.H.C.S., Halliwell B. (2017). Administration of Pure Ergothioneine to Healthy Human Subjects: Uptake, Metabolism, and Effects on Biomarkers of Oxidative Damage and Inflammation. Antioxid. Redox Signal..

[176] Cheah I.K., Halliwell B. (2012). Ergothioneine; antioxidant potential, physiological function and role in disease. Biochim. Biophys. Acta.

[177] Halliwell B., Cheah I.K., Tang R.M.Y. (2018). Ergothioneine-a diet-derived antioxidant with therapeutic potential. FEBS Lett..

[178] Halliwell B., Cheah I.K., Drum C.L. (2016). Ergothioneine, an adaptive antioxidant for the protection of injured tissues? A hypothesis. Biochem. Biophys. Res. Commun..

[179] Sakrak O., Kerem M., Bedirli A., Pasaoglu H., Akyurek N., Ofluoglu E., Gültekin F.A. (2008). Ergothioneine Modulates Proinflammatory Cytokines and Heat Shock Protein 70 in Mesenteric Ischemia and Reperfusion Injury. J. Surg. Res..

[180] Bedirli A., Sakrak O., Muhtaroglu S., Soyuer I., Guler I., Erdogan A.R., Sozuer E.M. (2004). Ergothioneine pretreatment protects the liver from ischemia-reperfusion injury caused by increasing hepatic heat shock protein 70. J. Surg. Res..

[181] Koay C., Selo M., Clerkin C., Ehrhardt C. (2019). In Vitro Studies on the Impact of Ergothioneine on Idiopathic Pulmonary Fibrosis Markers in Human Lung Epithelial Cells. Am. J. Respir. Crit. Care Med..

[182] Tang Y., Masuo Y., Sakai Y., Wakayama T., Sugiura T., Harada R., Futatsugi A., Komura T., Nakamichi N., Sekiguchi H. (2016). Localization of Xenobiotic Transporter OCTN1/SLC22A4 in Hepatic Stellate Cells and Its Protective Role in Liver Fibrosis. J. Pharm. Sci..

[183] Cheah I.K., Feng L., Tang R.M.Y., Lim K.H.C., Halliwell B. (2016). Ergothioneine levels in an elderly population decrease with age and incidence of cognitive decline; a risk factor for neurodegeneration?. Biochem. Biophys. Res. Commun..

[184] Smith E., Ottosson F., Hellstrand S., Ericson U., Orho-Melander M., Fernandez C., Melander O. (2019). Ergothioneine is associated with reduced mortality and decreased risk of cardiovascular disease. Heart.

[185] Koh S.S., Ooi S.C.-Y., Lui N.M.-Y., Qiong C., Ho L.T.-Y., Cheah I.K.-M., Halliwell B., Herr D.R., Ong W.-Y. (2020). Effect of Ergothioneine on 7-Ketocholesterol-Induced Endothelial Injury. Neuromol. Med..

[186] Cheah I.K., Halliwell B. (2020). Could Ergothioneine Aid in the Treatment of Coronavirus Patients?. Antioxidants.

[187] Xiao L., Zhao L., Li T., Hartle D.K., Aruoma O.I., Taylor E.W. (2006). Activity of the dietary antioxidant ergothioneine in a virus gene-based assay for inhibitors of HIV transcription. BioFactors.

